# Sustainability of global Golden Inland Waterways

**DOI:** 10.1038/s41467-020-15354-1

**Published:** 2020-03-25

**Authors:** Yichu Wang, Xiabin Chen, Alistair G. L. Borthwick, Tianhong Li, Huaihan Liu, Shengfa Yang, Chunmiao Zheng, Jianhua Xu, Jinren Ni

**Affiliations:** 1 The Key Laboratory of Water and Sediment Sciences, Ministry of Education, College of Environmental Sciences and Engineering, Peking University, Beijing, 100871 P. R. China; 2 Beijing Innovation Center for Engineering Science and Advanced Technology, Peking University, Beijing, 100871 P. R. China; 3 Institute for Infrastructure and Environment, School of Engineering, The University of Edinburgh, The King’s Buildings, Edinburgh, EH9 3JL UK; 4 National Inland Waterway Regulation Engineering Technology Research Center, Wuhan, 430010 P. R. China; 5 National Inland Waterway Regulation Engineering Technology Research Center, Chongqing Jiaotong University, Chongqing, 400074 P. R. China; 6 Center for Global Large Rivers, School of Environmental Science and Engineering, Southern University of Science and Technology, Shenzhen, 518055 P. R. China; 7 Department of Environmental Management, Peking University, Beijing, 100871 P. R. China

**Keywords:** Environmental sciences, Environmental social sciences

## Abstract

Sustainable inland waterways should meet the needs of navigation without compromising the health of riverine ecosystems. Here we propose a hierarchical model to describe sustainable development of the Golden Inland Waterways (GIWs) which are characterized by great bearing capacity and transport need. Based on datasets from 66 large rivers (basin area > 100,000 km^2^) worldwide, we identify 34 GIWs, mostly distributed in Asia, Europe, North America, and South America, typically following a three-stage development path from the initial, through to the developing and on to the developed stage. For most GIWs, the exploitation ratio, defined as the ratio of actual to idealized bearing capacity, should be less than 80% due to ecological considerations. Combined with the indices of regional development, GIWs exploitation, and riverine ecosystem, we reveal the global diversity and evolution of GIWs’ sustainability from 2015 to 2050, which highlights the importance of river-specific strategies for waterway exploitation worldwide.

## Abstract (web summary)

The exploitation of rivers has been at the detriment of river ecosystems. Here the authors propose a concept of Golden Inland Waterways (GIWs) to represent large waterways and find that the exploitation ratio threshold around the turning point for most GIWs appear to be less than 80%, subject to ecological constraints.

## Introduction

Inland waterways play an important role in the global transportation system^1,2^, but over-exploitation of waterways for navigational purposes^3^ has often been to the detriment of river ecosystems^4,5^. Each inland waterway has a bearing capacity, which is largely determined by local hydro-geomorphic conditions such as depth, width and velocity of river flow, and duration of freeze-over events^3^. Inland waterways are often modified to expand their bearing capacity^6^ in response to increasing transport need resulting from socio-economic development of the associated river basins. Such modifications may lead to changes in riverbed geomorphology^7^, and affect habitats of aquatic organisms as well as impair the functioning of the river ecosystem^8^. Moreover, maintenance dredging is usually necessary for waterway regulation, and requires sustained investment^6^. Therefore, overall costs become extremely high when restoring a river ecosystem, once ecological damage has occurred^9–11^.

Regional socio-economic development requires sustainable inland waterways for transporting goods and passengers in large river basins^12–14^. Bearing in mind that the essence of regional sustainability is to protect the environment while achieving socio-economic development goals^15–18^, the maintenance of river health is of particular importance in supporting the long-term provision of ecosystem goods, services, and values for future needs^19^. In other words, sustainable inland waterways, while expanding bearing capacity to meet the increasing transport need driven by regional development, must protect major ecological functions of river systems relevant to channel continuity, riparian and floodplain connectivity, flow regime, and biodiversity^20,21^. Long-term sustainability of inland waterways not only involves attaining consistency between bearing capacity and transport need but also requires a tradeoff between waterway exploitation intensity, infrastructure maintenance, and ecological conservation/restoration. In addition, climatic and hydrological uncertainty may pose further challenges to waterway sustainability^19,22^.

Here, we introduce the concept of a Golden Inland Waterway (GIW), which represents a large inland waterway with considerable bearing capacity and increasing transport need (or potential) driven by prosperous socio-economic development in its basin. A GIW could also be regarded as the main axis running through a large-river economic belt which acts as an important conveyor supporting regional sustainability. Previous studies of the sustainable development of inland waterway transport systems have been made at regional scale^12–14^ and so lack insight into the diverse sustainability of global waterways at different development stages. As emerging economies undergo rapid development^23,24^ such as in the cases of Brazil, Russia, India, China, and South Africa, there is usually an associated surge in demand for inland waterway transport, and so it is important to understand the sustainability of GIWs at different development stages and their implications for overall regional sustainability.

The most challenging task is to identify the threshold for GIWs exploitation under ecological considerations, which could be specifically quantified by establishing a set of indices to measure ecological pressures such as river fragmentation, wetland dis-connectivity, flow disruption, and loss of biodiversity^5,25–27^. Furthermore, eco-efficiency is another effective parameter used to measure regional sustainable development, which is evaluated according to multiple dividends arising from basic need, economic growth, resource conservation, and ecological protection^28^. For example, previous studies have adopted the ratio between economic performance (e.g. Gross Domestic Product, (GDP)) and environmental impact (e.g. ecological footprint) to evaluate regional eco-efficiency and to explore the decoupling effect of resource consumption, pollution emissions, and economic growth^29^. In light of accelerating stressors from economic development^30^, population growth^31^, and climate change^22^ in different parts of the world, the concept of GIWs should be very useful to inform river transport planning and regional development.

**Fig. 1:**
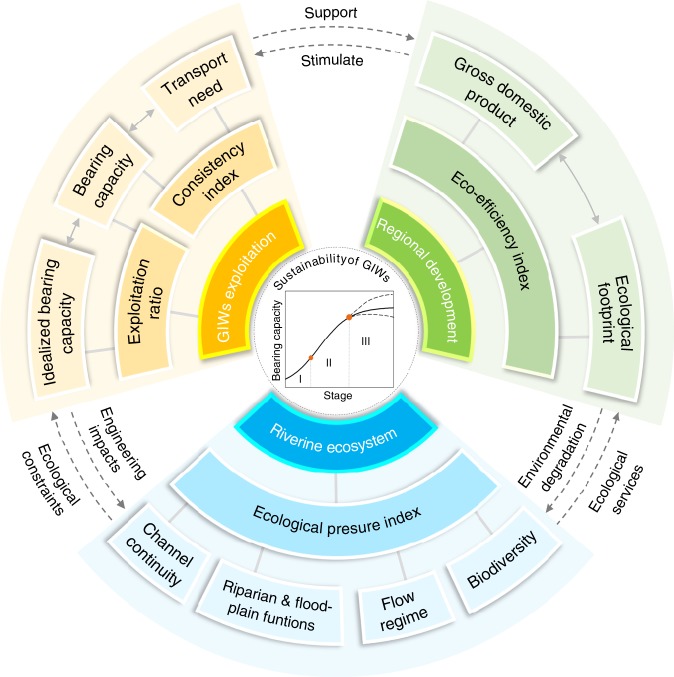
Hierarchical framework for assessing sustainability of global Golden Inland Waterways (GIWs). The framework integrates three primary sectors, i.e. GIWs exploitation, riverine ecosystem, and regional development. First, the stage of development for each of the GIWs is primarily determined from the regional development sector. Second, regional development would stimulate waterway transport need and require expansion in bearing capacity of specific GIWs. Third, the exploitation ratio is identified in the GIWs exploitation sector for the goal of regional development, but should not exceed a certain threshold due to ecological considerations. Fourth, ecological pressure from engineering practice is assessed in the riverine ecosystem sector to maintain the fundamental ecological services for regional development. Finally, sustainability of GIWs is estimated in terms of the metrics from the three sectors.

This paper identifies GIWs from 66 global large rivers (basin area > 100,000 km^2^). The development paths of GIWs worldwide are examined in terms of a general three-stage route with particular attention to the exploitation threshold due to ecological considerations in the vicinity of the turning point from the developing to the developed stage. Using a comprehensive framework (Fig. 1) to correlate data related to GIWs exploitation, riverine ecosystem, and regional development, we examine the sustainability of global GIWs in 2015 and 2050, which highlights the need for GIWs exploitation in the context of health of the local ecosystem and regional sustainability.

## Results and Discussion

### Identification and global distribution of GIWs

**Fig. 2:**
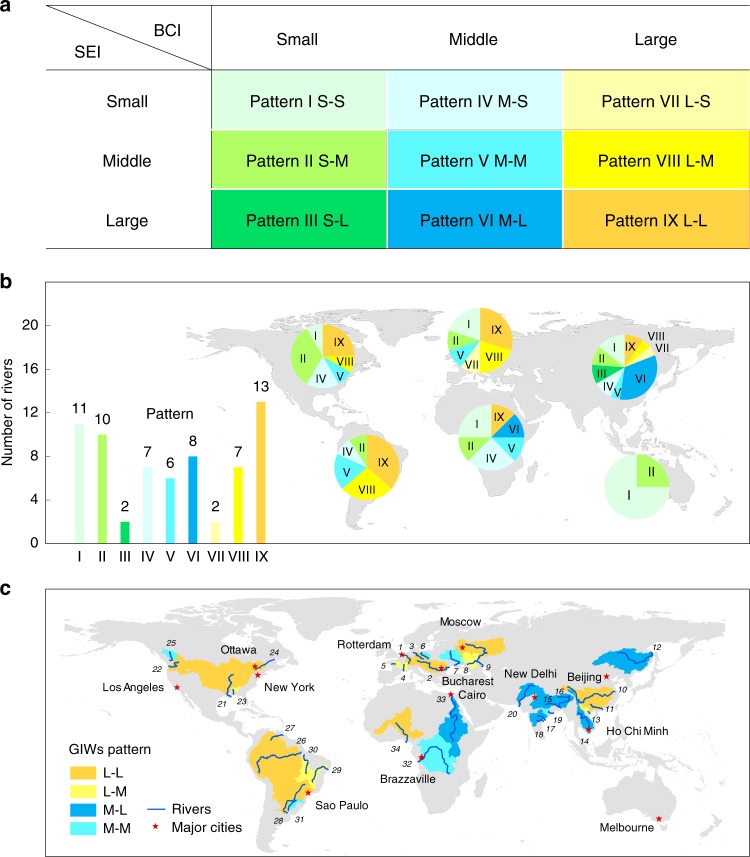
Identification and global distribution of Golden Inland Waterways (GIWs). **a** Nine patterns of inland waterway classified by bearing capacity index (BCI) and socio-economic index (SEI). BCI and SEI were each divided into three levels (small, S; middle, M; and large, L) at threshold values of 0.33 and 0.67 (for details of the thresholds see Methods). **b** Numbers of each pattern of inland waterway according to the foregoing pattern classification system, and their distribution in six continents, obtained for 66 large rivers worldwide. **c** Map of 34 GIWs, with patterns of M-M, M-L, L-M, and L-L. The GIW numbers marked in (**c**) coincide with those in Supplementary Tables 4 and 5. Source data are provided as a Source Data file.

Nine types of large waterways were identified (Fig. 2a and Supplementary Table 1) based on a bearing capacity index (BCI) (Supplementary Fig. 1), determined as the basin-averaged inland waterway bearing capacity (see Methods); and a socio-economic index (SEI) (Supplementary Fig. 2) established from GDP, agriculture and industry outputs, and population (see Methods). BCI and SEI were each divided into three levels (small S; middle M; and large L) at threshold values of 0.33 and 0.67, which were primarily determined according to the significance of cost-effective advantage of inland waterway transport^32^ and the level of human development of the river basin of interest^33^ (details see Methods). Consequently, nine basic patterns of inland waterway were classified as L-L, L-M, L-S, M-L, M-M, M-S, S-L, S-M, and S-S (the letters prior and post the hyphen denote the level of BCI and SEI for inland waterways, respectively). Figure 2b shows the global distribution of all the different types of waterways, of which four types, L-L, L-M, M-M, and M-L, were further identified as GIWs. The identified GIWs have a qualified freight volume to take low-cost advantage of inland waterway transport^32^, and a middle to high socio-economic development level to simulate transport need^33^. Figure 2c shows that the L-L type occurs mainly in Europe, the Americas, and Asia; the L-M type in Europe, North America and Asia; the M-M in South America and Europe; and the M-L mostly in Asia. No GIW is in Oceania. Three GIWs are observed in Africa, where countries are in the early or middle stage of industrialization, despite abundant natural resources and huge development potential. It should be noted that GIW is not an absolute concept and so the threshold used for its identification could be adjusted based on revised needs or further expert opinions.

### Characterization of GIWs’ development paths and stages

**Fig. 3:**
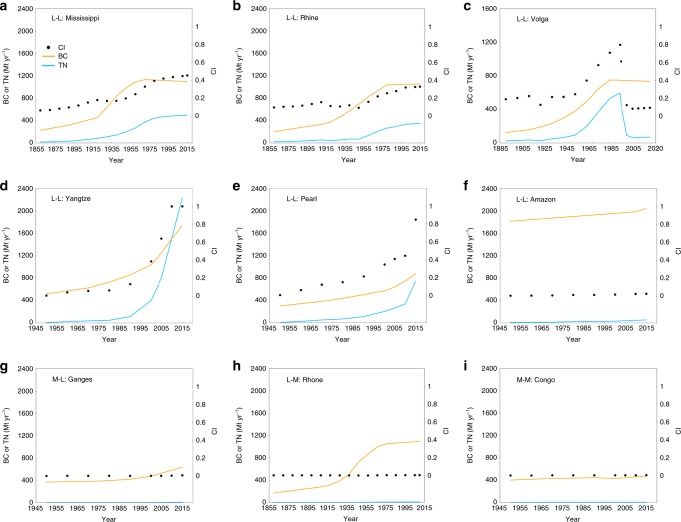
Development paths of nine representative Golden Inland Waterways. In each of the nine sub-graphs, dynamic changes are shown of bearing capacity (BC, Mt yr^−1^), transport need (TN, expressed as actual freight transport volume, Mt yr^−1^), and consistency index (CI = 0–1, defined as the ratio of TN to BC). These include (**a**–**f**) the L-L pattern represented by Mississippi, Rhine, Volga, Yangtze, Pearl, and Amazon, respectively; (**g**) the M-L pattern represented by Ganges; (**h**) the L-M pattern by Rhone; and (**i**) the M-M patter by Congo. Blue and yellow lines denote the evolution of TN and BC, respectively, whereas black dots indicate the trend of CI. Source data are provided as a Source Data file.

Figure 3 shows the development paths of nine representative GIWs expressed in terms of bearing capacity and transport need (given by freight transport volume, see Methods). We first consider L-L waterways. Figure 3a shows that the inland waterway bearing capacity of the Mississippi sharply increased between the 1930s and 1970s, when navigation improvement works were undertaken, and later declined as the waterway infrastructure aged^34^. The volume of freight passing through the Mississippi waterway increased almost exponentially until the 1980s, but then flattened off. The Rhine followed a similar development path (Fig. 3b)^35^. Conversely, transport need in the Volga (Fig. 3c) declined significantly from 595 Mt in 1989 to ~70 Mt in 2015, following the demise of the Soviet Union. In recent years, the Yangtze experienced an exponential growth in transport need, with freight volume reaching 2180 Mt in 2015, a value nearly five times higher than that in 2000 (Fig. 3d). Meanwhile, Yangtze’s bearing capacity also increased significantly to 1700 Mt in 2015. The Pearl River has experienced a similar development path (Fig. 3e), being situated close to a special economic zone in south China; its freight volume and bearing capacity were 737 Mt and 718 Mt in 2015. As the largest river in the world, the Amazon exhibited a remarkable discrepancy between its bearing capacity of 2039 Mt and transport need of 51.92 Mt in 2015 (Fig. 3f), which offers an opportunity for future increase in inland navigation.

Figure 3g–i shows the evolution of the remaining three classes of GIW. The Ganges is M-L, with large SEI (0.92) like the Yangtze (0.99). However, the Ganges has BCI of 0.63, much smaller than that of the Yangtze (0.97), owing to India’s monsoon climate and lower investment in waterway infrastructure. From the 1980s onwards, the bearing capacity of the Ganges increased to 614 Mt whereas its transport need rose only slightly to 3.92 Mt by 2015 (Fig. 3g). The L-M GIWs generally exhibited bearing capacity that exceeded development need over long periods (e.g. Rhone, Fig. 3h). The Congo (Fig. 3i), an M-M waterway, appears to have followed a similar development path to the Ganges; the bearing capacity of the Congo has grown to 460 Mt yr^−1^ far larger than its transport need about 1.5 Mt yr^−1^, offering a huge surplus potential for socio-economic development.

The foregoing illustrate the diverse development paths taken by typical GIWs, influenced by geographical, societal, and economic conditions. Taken overall, the GIW development path follows an S curve at a slow-fast-slow rate, with two turning points that separate the three development stages: initial, developing, and developed. These three stages are consistent with Chenery et al.’s theory^36^ in which industrialization is divided into six evolutionary phases. For each GIW, the development stage can be determined through the proportion of increase in agricultural, industrial and service industries as well as the GDP per capita (Supplementary Table 2).

### Consistency between bearing capacity and transport need

To promote a high level of potential socio-economic development, GIWs must achieve a proper balance between bearing capacity and transport need. However, these are frequently inconsistent because both undergo separate dynamic changes. The variation in coordination between transport need and bearing capacity was tracked using a consistency index (CI) defined as the ratio of freight transport volume to bearing capacity of inland waterways (see Methods) during different GIW development stages.

At the initial stage, a lower value of CI (<0.2) results from low social productivity and transportation need, as exemplified by the Ganges and the Congo (Fig. 3g, i) for which CI < 0.05. During the developing and developed stages, different industrialization and urbanization processes lead to diverse development modes. For example, the Mississippi and the Rhine (Fig. 3a, b) experienced considerable economic growth and moderate waterway exploitation, with CI ranging from 0.2 in the 1930s to 0.6 in the 1970s. Although certain GIWs, including the Amazon, are presently undergoing an economic boom, their CI is less than 0.05 (Fig. 3f) due to their immense bearing capacity. However, CI for the Volga increased to 0.8 during the developing period (1950s–1990s) but significantly decreased at the second developmental stage turning point due to a marked decline of transport need during the break up of the former Soviet Union. Afterwards, over-exploitation of its inland waterways driven by development inertia incurred unacceptable cost (Fig. 3c). The development path of the Volga serves as a warning that GIWs in rapidly developing regions, such as the Yangtze and the Pearl river basins, might also experience great challenges in the course of achieving a balance between increasing bearing capacity, ecological alteration, and socio-economic development. As illustrated in Fig. 3d, e, the CI of the Yangtze and the Pearl rapidly increased from ~0.1 to 1.0 from the 1980s to 2015; planners nevertheless contemplate further waterway expansion.

These empirical results show that the ideal range of CI for a long-term balance between bearing capacity and transport need seems to be in the range 0.2–0.6 for most GIWs at developing and developed stages, particularly those with similar development modes to those of the Mississippi and the Rhine. Values of CI that are too small (<0.2) or too large (>0.6) are both unsuitable for GIW development. Too small CI (<0.2) means that the potential and function of a GIW is far from fully developed. Too large CI (>0.6), impling a too tight pace between capacity and need, would lead to overload of inland waterways which restricts the efficiency and safety of shipping services. In this case, government usually tends to expand continuously the bearing capacity of inland waterways to address transport need and to enhance navigational safety^6^, which would greatly increase the risk of over-exploitation driven by development inertia, and excess capacity of inland waterways driven by Factor Hoarding theory^37^. Therefore, tracking the evolution of CI would be helpful to decision makers whose goal is to maintainning an appropriate pace of expansion of in bearing capacity of GIWs.

### GIWs exploitation and limitation

**Fig. 4:**
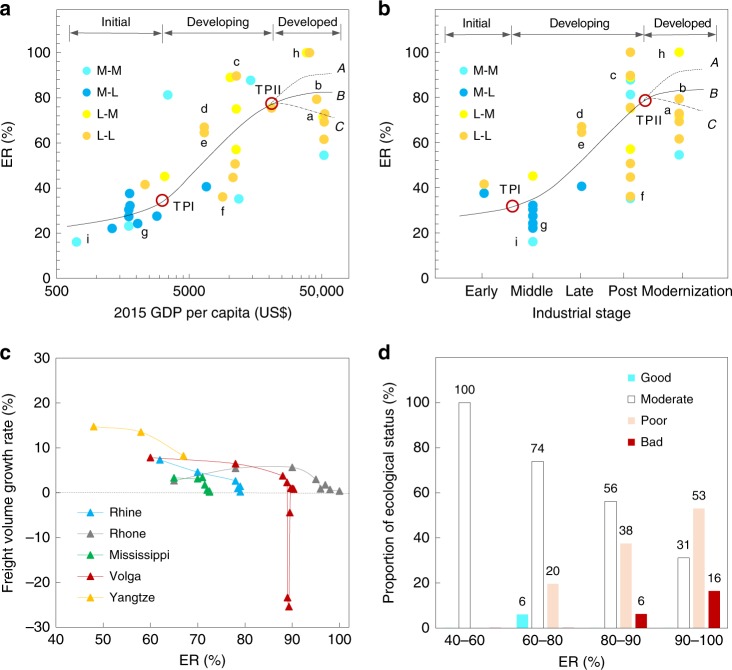
Exploitation ratios (ER) and threshold of representative Golden Inland Waterways (GIWs). ER (%) is the ratio of actual to idealized bearing capacity. **a**, **b** Basin-average ER of various GIWs (small letters a-i corresponding to the nine representative waterways in Fig. 3) at different development stages in terms of 2015 GDP per capita (in 2010 US$) and industrialization stage in 2015, respectively. The two turning points (TPI, TPII) separating the three stages are marked by red hollow circles. GIWs at the developed stage after the TPII show diverse ER (58–100%) as the consequences of different development strategies (A, radical; B, moderate; and C, conservative). **c** Freight volume growth rate (%) under varying ER for six typical GIWs. **d** Proportion of reaches with different levels of ecological status, corresponding to varying ER from 134 reaches of six European GIWs. Source data are provided as a Source Data file.

Exploitation intensity of inland waterways directly influences the bearing capacity of waterways and ecological stress on river basins. Exploitation ratio (ER), defined as the ratio of actual to idealized bearing capacity of inland waterways (see Methods), was used to examine the exploitation intensity of GIWs at different stages. The idealized bearing capacity, in the absence of navigation obstacles, may be determined from channel dimensions estimated from river discharge data^38^. Figure 4a, b shows the relationship between ER and development stage for all 34 GIWs in 2015. Following Chenery et al.^36^ (Supplementary Table 2), the GIW development stage may be interpreted through either GDP per capita (Fig. 4a, 2015 data based on 2010 US$) or industrial structure (Fig. 4b).

For GIWs at the initial stage, basin-averaged ER varied from 16% (Congo) to 45% (Red). At low ER during the initial stage, river ecological pressure is unlikely to arise from inland waterway construction. The first turning point, TPI, occurs at the transition from initial to developing stage (Fig. 4a, b), driven by increasing economic prosperity. The value of ER corresponding to TPI is imprecise, given different socio-economic development modes near the turning point, but is generally below 40%.

During the GIW developing stage, basin-averaged ER ranges from 35% (Uruguay) to 89% (Volga). GIWs in South America usually have relatively low ER (e.g. Amazon 36%, Orinoco 45%, and Parana 51%) due to their large bearing capacities. GIWs with higher ER are generally located in Europe and Asia (e.g. Don 89%, Oder 87%, Yangtze 67% and Pearl 65%). The second turning point, TPII, occurs at the transition from developing to developed stage (Fig. 4a, b). Challenges to GIW sustainability occur at TPII because of the different possible development strategies (e.g. A, radical; B, moderate; and C, conservative) (Fig. 4a, b) and thus influence long-term sustainability, given that the design life of inland waterway infrastructure usually exceeds 50 years^39^.

What value of ER is best for GIW sustainability about TPII? This can be answered from three perspectives. From the experience perspective, during the developed stage, the past expansion of GIWs suggests a maximum value of ER of about 80% is sensible (Fig. 4a, b), noting the average ER value for all GIWs in the developed stage is 79%. In practice, this threshold should be identified for regional goals and might be slightly different depending on ecological conditions; however, it should be noted that GIWs would hardly be sustained if ER were above 80%. From the economic perspective, considerable economic loss could occur when ER exceeds 80% in order to maintain exaggerated waterway capacity. In fact, sustained investment for regular maintenance of waterway infrastructure is still needed (see e.g.^6,40^, Supplementary Table 3) even if the high growth rate in freight volume begins to turn down (Fig. 4c). A pertinent lesson can be learned from the Volga River, where ER reached 89% as the freight volume growth rate became negative in the 1990s. From the ecological perspective, a greater risk of riverine ecological deterioration would be encountered when ER is over 80%. Figure 4d classifies the ecological status^41^ of 134 reaches in six European GIWs (i.e. Rhine, Danube, Elbe, Rhone, Loire, and Oder) into four grades (good, moderate, poor, and bad). The proportion of reaches with moderate status decreases from 100% to 31% with increasing ER; however, the proportion with poor and bad status increases significantly when ER exceeds 80%. Although other engineering schemes such as reservoirs, irrigation systems, and inter-basin transfer canals, may also impact on the health of a river ecosystem, over-exploitation of an inland waterway will lead inevitably to an unsustainable river ecosystem. Without doubt, ER can provide early warning of possible over-exploitation of GIWs and ecological consequences for river basins.

### Health of riverine ecosystems impacted by GIW exploitation

Engineering projects during waterway construction greatly influence structures and functions of river ecosystems from morphological, hydrological, and biotic perspectives^4,5,25–27^. An ecological pressure index (EPI) was introduced to evaluate the engineering impact on functionality of the river ecosystem, notably the key components of habitats such as channel, riparian, floodplain, and flow environments. Continuous river networks are fragmented by navigational lock-dam systems. Natural physical and biological interconnections between river channels and their floodplains are severed by river channel deepening and widening projects, and shoreline fortifications. Local riparian and floodplain habitats are degraded by channelization and bank hardening during waterway exploitation. The hydrological regimes of rivers alter due to the effect of navigational requirements on flow regulation. All these foregoing habitation alterations further influence the biodiversity of riverine ecosystem. Supplementary Fig. 3 summarizes the hierarchical system established to evaluate EPI, in which the health status of a riverine ecosystem impacted by waterway exploitation could be presented by a set of metrics (see Methods) including the river fragmentation index (FI), wetland dis-connectivity index (WDI), fraction of impervious surfaces (FIS), flow disruption index (FDI), fish richness index (FRI), and proportion of non-native fish (PNF).

**Fig. 5:**
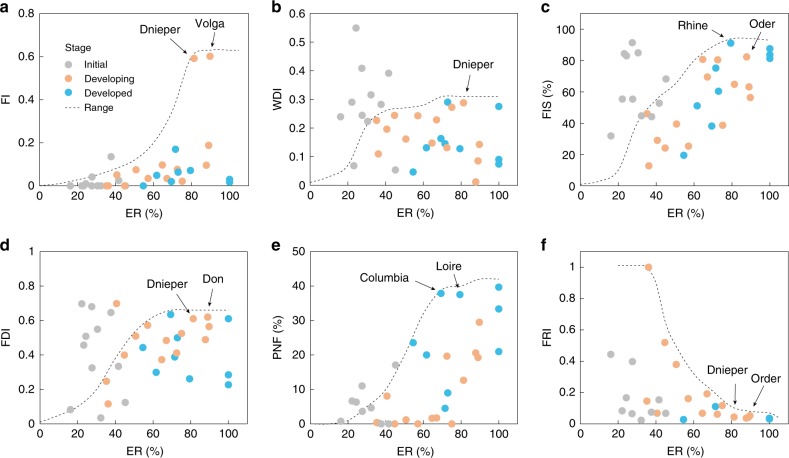
Ecological indices and thresholds under waterway exploitation. Relationship between: **a** Exploitation ratio (ER, %) and fragmentation index (FI). **b** ER and wetland dis-connectivity index (WDI). **c** ER and fraction of impervious surfaces (FIS, %). **d** ER and flow disruption index (FDI). **e** ER and proportion of non-native fish (PNF, %). **f** ER and fish richness index (FRI). The arrows indicate critical values of the metrics as ER approaches 80% presented by typical waterways. Source data are provided as a Source Data file.

In this system, ecological thresholds are defined as the critical conditions beyond which the key ecological functions of river ecosystem would be significantly damaged due to over-exploitation of GIWs (e.g. as ER approaches its threshold of 80%). Correspondingly, the ecological thresholds are identified as FI < 0.6, WDI < 0.3, FIS < 0.85, FDI < 0.65, FRI > 0.05, and PNF < 40%, respectively (Fig. 5).

The relationship between ER and EPI for 34 GIWs is displayed in Supplementary Fig. 4. For GIWs at the initial stage, most have a low value of EPI (<0.7) except Krishna, Ganges, and Indus. Ecological degradation of these three rivers might be due to human activities such as irrigation, hydropower generation, and drinking water abstraction, rather than inland waterway exploitation. For GIWs at the developing stages, EPI increases from 0.12 to 0.6 when ER changes from 35% to 75%. When ER > 80%, EPI of riverine ecosystems (e.g. Volga, Don, Oder, and Dnieper) increases significantly (0.57–0.83, Supplementary Fig. 4). The Volga and Dnieper are exposed to a high level of river fragmentation, which would further restrict migration of aquatic species within the river networks (Fig. 5a). The flow regimes of the Don and Dnieper are significantly disrupted (Fig. 5d), which might further alter hydrological regimes experienced by downstream aquatic organisms and facilitate invasion by lentic species. The most serious issue affecting the Oder seems to be the high fraction of impervious surface area (Fig. 5c), which would alter the channel morphology and degrade riparian habitats. Moreover, the Dnieper shows severe wetland dis-connectivity (Fig. 5b), and as a result, floodplain regions are likely to become dysfunctional. For GIWs at the developed stage (Supplementary Fig. 4), although EPI still increases with ER, EPI exhibits a relatively low value compared with GIWs at the developing stage, even for rivers with ER > 80% (e.g. Loire, Elbe, Rhone). One of the possible reasons is that large-scale ecological restoration is undertaken for intensely exploited GIWs at the developed stage. Taking the Rhone River as an example, the Rhone Restoration Project^42^, implemented since the early 1990s, successfully remedied ecological functions severely damaged by navigation and other human activities, recovering minimum flows by a factor up to 10 and reconnecting about 50% of the floodplains to the main channel.

### Eco-efficiency of GIWs-affiliated basin

Eco-efficiency index (EEI), defined as the ratio of GDP to ecological footprint (see Methods), was used to measure socio-economic-ecological quality of the GIW-affiliated basins. As a macroscopic metric of regional development, EEI is expected to be maximized at a certain development stage in the GIW-affiliated basins.

Supplementary Fig. 5 illustrates the relationship between ER and EEI for GIW-affiliated basins at different stages. At the initial stage, EEI has a low value, ranging from 781 to 2146 US$ per gha, which is primarily due to insufficient local socio-economic development. At the developing stage, EEI ranges from 1595 to 5399 US$ per gha when ER is less than 80%. For ER > 80%, EEI decreases significantly (1122–3122 US$ per gha) due to increases in environmental degradation and resources consumption. At the developed stage, EEI exhibits a much higher value (6065–9756 US$ per gha), even for rivers with ER > 80% (e.g. Loire, Elbe, Rhone). This might be partially explained by the Environmental Kuznets Curve hypothesis^43,44^, i.e. as actual per capita income improves, investment in ecological restoration would ameliorate environmental quality (see e.g.^42^).

### Sustainability of GIWs in 2015 and 2050

To assess the long-term sustainability of global GIWs in 2015 and 2050, we propose a sustainability index (SI) which is a composite quantification based on scores of CI, ER, EPI, and EEI (see Methods). Maximization of EEI and minimization of EPI are two targets of GIWs sustainability in the context of economic growth and ecological health. Unity-normalization of the ascending rank order of data was used to evaluate the score of EEI and EPI over all basins (see Methods). Considering the nonlinearity of the constraints to sustainability, a normal distribution was used to evaluate the scores of CI and ER (Supplementary Fig. 6), with a preferred range of 0.2 < CI < 0.6 and an upper limit of ER = 80%. The SI metric provides an integrated measure of the sustainability of the GIWs required by regional development and ecosystem health (Fig. 1).

**Fig. 6:**
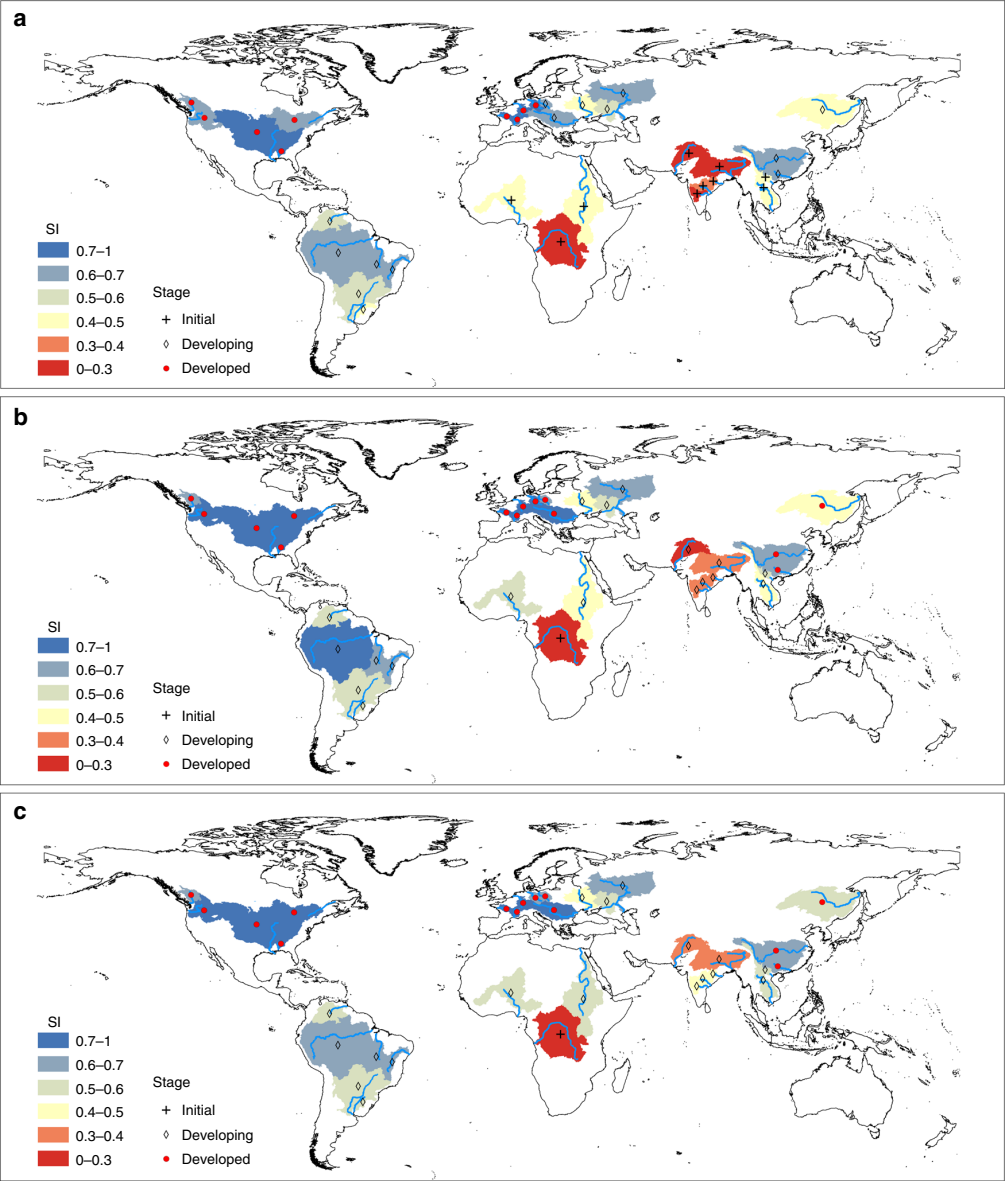
Global distribution of sustainability index (SI) and corresponding development stage of Golden Inland Waterways (GIWs) in 2015 and 2050. **a** SI of global GIWs in 2015. **b** SI of global GIWs in 2050 under the ER invariant scenario. **c** SI of global GIWs in 2050 under the scenario where adjustments are made to ER aiming at improving sustainability. Red-to-blue gradient indicates the increasing SI of GIWs. GIWs are considered as achieving low, middle, and high levels of sustainability when SI < 0.5, 0.5 < SI < 0.7, and SI > 0.7. Source data are provided as a Source Data file.

In 2015, a relatively low SI (<0.5) is derived for GIWs at initial stage of development in Asia and Africa (Fig. 6a) due to lower CI, ER as well as EEI, which implies less pressure from waterway exploitation at present but does not mean long-term sustainability at the developing and developed stages (Supplementary Tables 4 and 5). A moderate level of SI (0.5–0.7) is observed for GIWs at the developing stage, except for the Dnieper (SI = 0.46) and Amur (SI = 0.45). The Dnieper river basin is exposed to a very high threat of ecological deterioration (EPI = 0.83) caused by over-exploitation (ER > 80%) of its inland waterway, leading to low SI (Supplementary Table 5). Similar over-exploitation has also occurred in the Volga, Don, and Oder river basins (0.59 < SI < 0.61). For the Yangtze, Pearl, Danube, and Sao Francisco waterways (0.61 < SI < 0.7) whose ER values exceed 60%, there is an alarming risk of over-exploitation driven by development inertia. Meanwhile, the Yangtze and Pearl River basins exhibit a very low EEI (1595 US$ per gha), implying the necessity of industrial transformation (Supplementary Table 5). The remaining GIWs distributed in South America have moderate SI with smaller ER (e.g. Amazon, Parana, and Orinoco) due to their large idealized bearing capacity. For the foreseeable future, these waterways are likely to continue to meet long-term transport needs without requiring new infrastructure. All nine GIWs at the developed stage exhibit high sustainability (SI ≥ 0.7), and are distributed in Europe and North America. Exemplars of development paths are given by those followed by the Mississippi waterway (SI = 0.9) and Rhine waterway (SI = 0.93), with ideal CI (0.2–0.6) and ER (<80%), and relatively low EPI as well as high EEI. Although the Rhone, Loire, and Elbe waterways have low CI (0.001–0.025) and extremely high ER (~100%), they nevertheless achieve high sustainability due to their large score of EEI and EPI resulting from large-scale ecological restoration^42^.

By 2050, we estimate 10 GIWs will enter the developing stage (e.g. Ganges, Mekong, and Niger), and 5 GIWs (e.g. Danube and Yangtze) the developed stage (Supplementary Table 4) based on the predicted GDP per capita. Using linear regression, we also forecast the transport need expressed by freight transport volume in 2050 (Supplementary Table 4). Two scenarios were used to examine the possible changes to the sustainability of global GIWs by 2050: one where ER is kept constant; the other where hypothetical adjustments are made to ER of GIWs, and hence the bearing capacity and the waterway exploitation-induced ecological pressure also change (see Methods). For the first scenario, when ER is maintained at 2015 level (Fig. 6b), the SI values of the Ganges, Red, Amazon, Krishna, and Niger increase considerably (by 11–21%) in 2050; whereas the SI value for the Mekong decreases by 19% due to too large CI but a low EEI which implies a need to upgrading the waterway (Supplementary Table 5). The SI value of the remaining GIWs appears to be stable (relative percentage < 10%), confirming that ER is a key factor influencing the sustainability of GIWs. In the second scenario, the resulting level of sustainability of global GIWs in 2050 (Fig. 6c) is significantly improved compared both to the first scenario (Fig. 6b) and to the level of sustainability in 2015 (Fig. 6a). A significant increase in SI (by 10–50%) is obtained for 13 GIWs which are mainly distributed in south Asia and Africa (Supplementary Table 5). Furthermore, the Mekong, Red, Niger, Uruguay, Nile, and Amur waterways attain moderate sustainability, with SI exceeding 0.5. However, the intensity of economic development might place considerable pressure on these river ecosystems.

It is likely that climate change will have different impacts on different GIWs sustainability depending on their regional location. For GIWs, water depth is most sensitive to climate change. Droughts could severely affect navigational services though reducing low water levels either to completely non-navigable depths or to levels that freight volumes of vessels have to be reduced, resulting in increased transport prices and decreased welfare^22,45^. Floods threaten navigational safety and speed especially when water level exceeds a critical permitted threshold determined by infrastructure^45^. Herein, water depth data for GIWs are either provided by relevant government agencies or estimated from river discharges using a standard power law relationship. Further studies are recommended to obtain insights into climate impacts on GIWs sustainability by use of global circulation models, downscaling hydro-meteorological parameters to regional scale, and assessment of non-stationary statistical changes. Uncertainties and errors in estimates of river discharges introduced by projection of runoff to river discharge under climate change through either process-driven or data-driven models also merit careful analysis. For GIWs at high latitude, the annual navigable days influenced by ice formation might be another concern. However, the impacts of ice are limited considering its freeze-up duration or frequency, and are expected to reduce further because the projected temperature will increase in the future^45^.

### Implications for sustainable development of GIWs

The comprehensive framework for assessing GIWs sustainability (Fig. 1) is capable of communicating interactions among disparate data by providing links between regional socio-economic development, GIWs exploitation, and human pressure on the riverine ecosystem. In particular, the underlying metrics enable different options to be prioritized and respectively implemented, postponed, or even discounted according to expert judgement, which should be useful to decision makers concerned with basin-wide economic development and ecological restoration. A sensible way of undertaking this is to recommend strategies according to the state of development of the river basin under consideration.

For a GIW at initial stage of development, the GIW has insufficient transport need due to low socio-economic development level. With emerging socio-economic development, transport need is stimulated and waterway regulation projects are required to expand GIW bearing capacity through improved waterway conditions, suggesting increases in CI, ER, and potentially EPI. As GIWs transform from the initial to the developing stage in the forthcoming decades, planners should implement strategies that are not too conservative in order to exploit socio-economic opportunity.

During the developing stage, planners should attempt to achieve an optimal waterway exploitation ratio to address challenges to sustainability. In practice, for a GIW with ER < 60%, a minor increase in ER is recommended in the following decades. For a GIW where 60% < ER < 80%, the risk of over-exploitation driven by development inertia should be reduced, perhaps by lowering the gradient in ER with time. For an over-exploited GIW with ER > 80%, it is necessary to reduce the EPI through ecological restoration activities.

For GIWs at the developed stage, the aim should be to maintain the high value of SI. For a GIW with high ER, all that is required is to continue investment in waterway maintenance and ecological rehabilitation projects, and/or upgrading the quality (e.g. incorporation of multiple targets including recreation and ecology, and reassessment of transport need^3,46^) of the entire waterway system. In this case, the EPI metric is particularly important for monitoring purposes.

In practice, analysis of the metrics would be a rather more complicated exercise than indicated above because the target values would be necessarily case-specific, the processes underlying the metrics may interact, and detailed adjustment of sub-metrics may be required.

In the forthcoming decades, certain GIWs will experience adjustments in development path, and long-term strategy targeting sustainability is of particular significance. From the global perspective, our estimates of sustainability of GIWs highlight the importance of river-specific strategies for waterway exploitation in the context of regional development and ecological restoration.

## Methods

### Identification of GIWs

GIWs were quantitatively identified from 66 large inland waterways of basin area > 100,000 km^2^, with two variables characterizing their bearing capacity and transport need driven by socio-economic development within the basins: bearing capacity index and socio-economic index. Given that the scale varies by several orders of magnitude across different waterways, we used rank-normalization to reduce the relative influence of the indexes. The ranked indicator values were then normalized to unity (i.e. ranging from 0 lowest to 1 highest ranked river) in order to reduce distortion that would otherwise be introduced by low-valued raw indicators obtained for certain waterways. Information on the waterways was extracted from the global river network supplied by PKU^47^ and by HYDROSHEDS (http://www.hydrosheds.org/).

### Bearing capacity index

The bearing capacity index, BCI, was used to represent the navigational capacity of a given waterway. Inland waterway bearing capacity (BC) was approximated by the theoretical annual freight volume that can pass through a given waterway cross-section, determined from

1 $${\mathrm{BC}} = \frac{{{\it{MT}}}}{{K_{\mathrm{h}}}}q_{\mathrm{h}},$$

where *M* is the average tonnage (t); *T* is the number of navigable days per year; *K*_h_ is a design hourly factor (the ratio of design hourly traffic volume to annual average daily traffic volume, noting the heterogeneity of river traffic flow) whose value was set to a default of 0.14 owing to a lack of measured data; and *q*_h_ is the hourly basic inland waterway traffic capacity obtained from following equation which satisfies the bidirectional continuous traffic hypothesis:

2 $$q_{\mathrm{h}} = m_{\mathrm{u}}\frac{{3600{\,}\left( {v_{\mathrm{u}} - v_{\mathrm{w}}} \right)}}{{l_{\mathrm{u}}}} + m_{\mathrm{d}}\frac{{3600{\,}\left( {v_{\mathrm{d}} + v_{\mathrm{w}}} \right)}}{{l_{\mathrm{d}}}},$$

where *m*_u_, *m*_d_ are the numbers of upstream and downstream ships; *v*_u_, *v*_d_ are the upstream and downstream vessel speeds; *v*_w_ is the waterway flow velocity; and *l*_u_, *l*_d_ are the longitudinal domain lengths of upstream and downstream ships, estimated using a ship domain model^48^.

Basin-average bearing capacities were derived from the reach-scale bearing capacities through length-weighted averaging.

The normalized bearing capacity index (BCI) was given by

3 $${\rm{BCI}}_w = \frac{{\widehat {{\rm{BC}}}_w - \min \left( {\widehat {{\rm{BC}}}_w} \right)}}{{\max \left( {\widehat {{\rm{BC}}}_w} \right) - \min (\widehat {{\rm{BC}}}_w)}},$$

where $\widehat {{\mathrm{BC}}}_w$ is the ascending rank order over all waterways of bearing capacity at waterway basin *w*.

We assumed that the same type of vessel passes through the same grade of waterway wherever in the world. The average tonnage (*M*) of inland vessels (Supplementary Table 6) was estimated based on waterway grade determined by minimum waterway maintenance depth. As an approximation, we evaluated the grade of global waterways using the navigation standard of inland waterways of China. Minimum waterway maintenance depth of the 66 global inland waterways (Supplementary Table 7) was obtained from relevant government agencies (Supplementary Table 8). The annual navigable days (*T*) for each waterway with high latitude was estimated using data on freeze-up duration (see Supplementary Table 9) with *T* for the remaining inland waterways set to 0. Herein, *m*_u_ and *m*_d_ were set to 1; *v*_u_ and *v*_d_ were set to be 3–5 m s^−1^ and 5–7 m s^−1^; *v*_w_ was set to 1 m s^−1^.

Supplementary Table 6 also lists the values of *l*_u_ and *l*_d_. It should be noted that bearing capacity referred to the actual bearing capacity of inland waterways based on the actual minimum waterway maintenance depth. Further details of the reach-scale bearing capacity of global large inland waterways are given in Supplementary Fig. 7, and values of the BCI for each large river are listed in Supplementary Table 1.

### Socio-economic index

The socio-economic index (SEI) approximately represents transport need driven by socio-economic development, and was established from the GDP, agriculture and industry outputs (AIO), and population (POP), as follows:

4 $${\mathrm{SEI}}_w = \left[ \begin{array}{l}\frac{{\widehat {{\mathrm{GPD}}}_w - {\mathrm{min}}\left( {\widehat {{\mathrm{GDP}}}_w} \right)}}{{{\mathrm{max}}\left( {\widehat {\mathrm{GPD}}_w} \right) - {\mathrm{min}}\left( {\widehat {{\mathrm{GDP}}}_w} \right)}} \\ + \frac{{\widehat {{\mathrm{AIO}}}_w - {\mathrm{min}}\left( {\widehat {{\mathrm{AIO}}}_w} \right)}}{{{\mathrm{max}}\left( {\widehat {{\mathrm{AIO}}}_w} \right) - {\mathrm{min}}\left( {\widehat {{\mathrm{AIO}}}_w} \right)}} \\ + \frac{{\widehat {{\mathrm{POP}}}_w - {\mathrm{min}}\left( {\widehat {{\mathrm{POP}}}_w} \right)}}{{{\mathrm{max}}\left( {\widehat {{\mathrm{POP}}}_w} \right) - {\mathrm{min}}\left( {\widehat {{\mathrm{POP}}}_w} \right)}}\end{array} \right]/3,$$

where $\widehat {{\mathrm{GDP}}}_w$, $\widehat {{\mathrm{AIO}}}_w$, and $\widehat {{\mathrm{POP}}}_{i,w}$ are the ascending rank orders over all waterways of the three indicators, and *w* refers to a given waterway. Given the lack of statistical data on GDP, AIO, and POP at global basin scale, we used a partition coefficient matrix to estimate basin parameters from the datasets at country scale. Historical GDP, AIO, and POP data were all obtained from the United Nations database (http://data.un.org/) in the time period from 1970 to 2017. Supplementary Figs. 8–10 present the normalized GDP, POP, and AIO indices for global large inland waterways. Supplementary Table 1 lists the corresponding SEI for each large river.

We assumed equal weights in calculating SEI. Of course, it is extremely difficult to determine proper values for the weights owing to limited knowledge of the relative importance of each indicator. To test for sensitivity, we employed a Monte Carlo approach to simulate the effect of different weight scenarios on SEI. This approach generated random index weights between 0 and 1, assuming a uniform distribution, and we calculated the standard deviation of 10,000 simulation SEI results as the error using an equal weight hypothesis. We found SEI was not very sensitive to index weights for 76% of the 66 large rivers, with the relative difference ranging from −40% to 40% (Supplementary Fig. 11). Only a few rivers with very high GDP or population scores (e.g. Murray-Darling, Columbia, Congo, and Zambezi) displayed a relatively significant variation with the index weights.

### Extraction of GIWs

GIWs have comparative advantages in terms of both bearing capacity and transport need or potential. Therefore, we established a two-dimensional approach given by BCI and SEI in order to identify GIWs. BCI and SEI were each divided into three levels (large L, middle M, and small S) by certain thresholds; hence, nine basic patterns of inland waterway were classified as L-L, L-M, L-S, M-L, M-M, M-S, S-L, S-M, and S-S (the letters before and after the hyphen denote the level of BCI and SEI for inland waterways, respectively).

We define a GIW as an inland waterway with BCI and SEI simultaneously exceeding prescribed thresholds. The BCI threshold was determined based on average tonnage of ships. Previous experience suggests that the low-cost advantage of inland waterway transport starts to appear once the average tonnage of ships exceeds 300 t (corresponding to BCI = 0.29–0.34) and becomes significant when the average tonnage of ships exceeds 1000 t (corresponding to BCI ~ 0.62)^32^. The SEI threshold was determined according to the human development index (HDI) of the river basin of interest. HDI is a metric used to assess the social and economic development levels of countries or regions, and quantifies life expectancy, educational attainment, and income as a standardized number^33^. The median values of SEI corresponding to low human development basins (HDI < 0.55) and mid-to-high human development basins (0.55 < HDI < 0.8) are 0.28 and 0.64, respectively.

For simplicity, the lower band of equipartition of the normalized indices, 0.33, was set as a threshold value for both BCI and SEI used to identify GIWs (as M-M, L-M, L-M, and L-L patterns) for large rivers. The upper band, 0.66, was used as an approximate threshold for further screening the most representative GIWs (L-L pattern). It should be noted that GIW is not an absolute concept and so the threshold used for its identification is not a constant, but can be adjusted following expert opinion. When the threshold for identification of GIWs is varied, the number of GIWs changes accordingly. For example, by varying the threshold values by ±50%, we find that the number of identified GIWs changes from 34 for the baseline case to 28–41 (see Supplementary Table 10).

This approach not only reflects the comparative advantages of GIWs but also reveals the contradiction between existing inland waterway capacity and potential transport need driven by socio-economic development.

### Evaluation of sustainability of GIWs

Four indicators were used to evaluate the sustainability of GIWs: consistency index (CI), exploitation ratio (ER), ecological pressure index (EPI), and eco-efficiency index (EEI).

### Consistency between bearing capacity and transport need

The coordination (or gap) between navigability and transport need of GIWs was measured by a consistency index, defined as the ratio of transport need to bearing capacity. Given the substantial difference that can occur between magnitude of capacity and need of a given waterway, a normalized approach was taken as follows. If capacity > need, the consistency index CI_*i*,*w*_ in year *i* at waterway *w* was determined from

5 $${\mathrm{CI}}_{i,w} = \frac{{{\mathrm{TN}}_{i,w}}}{{{\mathrm{BC}}_{i,w}}}$$

in which BC_*i*,*w*_ is the bearing capacity in year *i* of waterway *w*, Mt yr^−1^; and TN_*i*,*w*_ is the transport need in year *i* of waterway *w*, Mt yr^−1^. If capacity ≤ need, CI_*i*,*w*_ = 1.0.

The basin-average consistency index (CI) was estimated from the basin-average transport need divided by the basin-average bearing capacity. Supplementary Fig. 12 shows the CI of global inland waterways in 2015. More details see Supplementary Table 5.

The transport need (TN) of GIWs was quantified by the freight transport volume (Supplementary Table 11). We applied an elastic coefficient method to estimate the historical and future freight volumes of representative GIWs; the projection outcome obtained using this method closely matched the aggregated result of detailed transportation forecast models, such as TRANS–TOOLS^49^. The compound annual growth rate (CAGR) of freight volume was estimated from:

6 $${\mathrm{CAGR}}_{{\mathrm{freight}}} = {\mathrm{EC}} \times {\mathrm{CAGR}}_{{\mathrm{GDP}}},$$

where CAGR_freight_ is the compound annual growth rate of freight volume, EC is the elastic coefficient estimated for different scenarios (Supplementary Table 12), and CAGR_GDP_ is the compound annual growth rate of GDP. We used historical and future GDP data from Maddison Project Database (https://www.rug.nl/ggdc/historicaldevelopment/maddison/releases/maddison-project-database-2018) and International Futures (IFs) platform Version 7.31 produced by the University of Denver (https://pardee.du.edu/) to calculate CAGR_GDP_ over ten year intervals. Future population, and industrial and agricultural output data were also derived from the International Futures (IFs) platform. And the historical bearing capacity of typical GIWs was estimated from waterway maintenance dimensions data available for particular years, including the start and end times of large-scale waterway regulation projects.

### GIWs exploitation ratio

The exploitation ratio describing the exploitation intensity of GIW *w* at reach *l* was estimated from

7 $${\mathrm{ER}}_{l,w} = \frac{{{\mathrm{BC}}_{l,w}}}{{{\mathrm{IBC}}_{l,w}}},$$

where BC_*l*,*w*_ is the bearing capacity of waterway *w* at reach *l*; and IBC_*l*,*w*_ is the idealized bearing capacity of waterway *w* at reach *l*. The basin-average exploitation ratio (ER) was finally estimated from the basin-average bearing capacity divided by the idealized basin-average bearing capacity.

The idealized bearing capacity (IBC) represents the maximum potential of bearing capacity for an inland waterway, and can also be estimated from Eqs. (1) and (2). The only difference is that minimum waterway maintenance depth is replaced by river depth (*d*_w_) using

8 $$d_{\mathrm{w}} = {\mathrm{1}}{\mathrm{.5}}d_{{\mathrm{dry}}},$$

where *d*_dry_ is the average depth in the dry season estimated from the river discharge by power law relationships^38^. Considering the potential of exploitation and the relationship between average depth and fairway maintenance depth, we employed an amplification factor to calculate idealized fairway depth. After the grade of waterway was specified, the idealized bearing capacity was calculated using Eqs. (1–2). Supplementary Fig. 13 and Supplementary Fig. 14 separately display the idealized bearing capacity (IBC) of global large rivers and reach-scale exploitation ratio (ER) of global GIWs in 2015.

### Ecological pressure index

The health of a river ecosystem affected by human activities is measured through EPI, which was evaluated as

9 $${\mathrm{EPI}} =	 \,\, \frac{{{\mathrm{FI/FI}}_0 + {\mathrm{WDI/WDI}}_0 + {\mathrm{FIS/FIS}}_0}}{3}\\ 	{\,}+ \frac{{{\mathrm{FDI}}}}{{{\mathrm{FDI}}_0}} + \frac{{\left( {{\mathrm{1 - FI}}} \right)/\left( {{\mathrm{1 - FI}}_0} \right) + {\mathrm{PNF/PNF}}_0}}{2},$$

where FI, WDI, FIS, FDI, FRI, and PNF are the fragmentation index, wetland dis-connectivity index, fraction of impervious surfaces, flow disruption index, fish richness index, and proportion of non-native fish; FI_0_, WDI_0_, FIS_0_, FDI_0_, FRI_0_, and PNF_0_ are threshold values of the foregoing indicators when ER approaching 80%. It should be noted that FI is calculated using Eq. (10), noting that not all dams are built for navigability purposes,

10 $${\mathrm{FI}} = {\mathrm{FI}}^{\prime} \times \alpha,$$

where *α* is a proportionality factor determined from

11 $$\alpha = \frac{{N_{{\mathrm{navi}}}}}{{N_{{\mathrm{total}}}}}$$

in which *N*_navi_ is the number of dams used for navigability in a basin, and *N*_total_ is the total number of dams in a basin. FI’, WDI, FIS, and FDI data were extracted from http://www.riverthreat.net/data.html. *N*_navi_, and *N*_total_ were obtained from Global Reservoir and Dam (GRanD) Database (http://globaldamwatch.org/grand/). Data on the total number of freshwater fish species living in the river basin and the number of non-native fish species were obtained from the Fish-SPRICH database (https://static-content.springer.com/esm/art%3A10.1007%2Fs10750-012-1242-6/MediaObjects/10750_2012_1242_MOESM2_ESM.txt).

Supplementary Table 5 lists the EPI for each GIW in 2015.

### Eco-efficiency index

Eco-efficiency implies increased output that satisfies human demand, low resource consumption, and minimal environmental impact. In a sense, eco-efficiency represents the level of ecological civilization (where humans repair previous ecological damage and integrate properly with nature) of a region. For each GIW, an eco-efficiency index (EEI) in US$ per gha can be determined from^29^

12 $${\mathrm{EEI}} = \frac{{{\mathrm{GDP}}}}{{{\mathrm{EF}}}},$$

where GDP (in US$) is the gross domestic product and EF (in global hectares, gha) is the ecological footprint of the GIW.

The ecological footprint (EF) representing resource consumption is a measure of how much area of biologically productive land or water an individual, population or activity requires to produce all the resources it consumes and to absorb the waste it generates^50,51^. GDP data were extracted from the United Nation database (http://data.un.org/). Ecological footprint data were obtained from the Global Footprint Network (https://www.footprintnetwork.org/). Further details on the calculation are given by Lin et al.^51^. Supplementary Table 5 lists the EEI for each GIW in 2015.

### Sustainability of GIWs

Sustainability of GIWs was evaluated by means of a sustainability index (SI) based on the scores of a consistency index (*S*_CI_), an exploitation ratio (*S*_ER_), and a score of EPI and EEI (*S*_EEI, EPI_). Neither CI nor ER are monotone functions with respect to sustainability. Therefore, we used a normal distribution to evaluate the scores of CI and ER from

13 $$S_{{\mathrm{CI}}} = \frac{1}{{\sqrt {2{\uppi}} \sigma }}{\mathrm{e}}^{ - \frac{{{\mathrm{CI}} - \mu }}{{2\sigma ^2}}},$$

where *S*_CI_ is the score of CI, *μ* = 0.6, and *σ* = 0.4, and

14 $$S_{{\mathrm{ER}}} = \frac{1}{{\sqrt {2{\uppi}} \sigma }}{\mathrm{e}}^{ - \frac{{\frac{{{\mathrm{ER}}}}{{100}} - \mu }}{{2\sigma ^2}}},$$

where *S*_ER_ is the score of ER, *μ* = 0.8, and *σ* = 0.4. These equations are plotted in Supplementary Fig. 6.

*S*_EEI,EPI_, the score of EEI and EPI, was evaluated using a similar equation to Eq. (4) using data on the combination of EPI and EEI as ($\frac{{{\mathrm{EEI}}}}{{{\mathrm{1 + EPI}}}}$) for the year of interest. Here, SI is equal to the average of *S*_CI_, *S*_ER_ and *S*_EEI,EPI_. Results from sensitivity analysis for SI performed by Monte Carlo approach are displayed in Supplementary Figs. 15–17.

### Scenario analysis

Scenario analysis was used to forecast the sustainability of global GIWs in 2050. In the first scenario, ER for each waterway was kept constant at the 2015 value and changes only occur in the freight transport volume and EEI (See Supplementary Tables 4 and 5). The freight transport volume and EEI in 2050 were estimated by an elastic coefficient method using Eq. (6).

In the second scenario, ER was varied according to suggested measures aimed at improving sustainability. In this case, it was assumed that ER values of developing stage GIWs which underwent rapid development by 2015 (e.g. Yangtze and Pearl) should not exceed 80%, whereas ER values of GIWs undergoing more moderate development (e.g. Amazon and Tocantins) should be increased slightly (by no more than 10%). For GIWs that were in the initial stage in 2015, ER was permitted to increase more significantly (but by no more than 20%). For GIWs with ER higher than 80% in 2015, it was assumed that expansion had ended. The second scenario was idealized, in that BC and EPI also changed as ER varied (See Supplementary Tables 4 and 5).

For both scenarios, EEI values in 2050 were estimated through linear extrapolation of EEI data obtained during 2000–2014. EPI values in 2050 were estimated using the following regression formula for GIWs at the developing and developed stages, obtained from data in 2015,

15 $${\mathrm{EPI}} = 0.16 + 0.57{\mathrm{ER}}\left( {R^2 = 0.56} \right).$$

The bearing capacity of each waterway in 2050 was calculated from BC = ER × IBC, with IBC assumed unchanged.

Supplementary Table 13 provides a description of each metric mentioned in the Methods, along with their data source(s) and interpretation.

### Reporting summary

Further information on research design is available in the Nature Research Reporting Summary linked to this article.

## Supplementary information

Supplementary Information

Reporting Summary

## Data Availability

Data on the physical and socio-economic characteristics of global large inland waterways at reach scale are available from figshare [10.6084/m9.figshare.11653281]^52^. Basin-scale data related to inland waterways reported in this paper are provided in the Supplementary Information file and Source Data file. All other data, including river networks, basin boundaries, GDP, agriculture and industry outputs, population, river depths, dam distribution, ecological indices and ecological footprint are publicly available, as described in the Methods. The source data underlying Fig. 2‒6 and Supplementary Figs. 1, 2, 4, 5 and 7‒17 are provided as a Source Data file.

## Source data

Source Data

## Code availability

Python codes used (1) to estimate basin-scale parameters from the datasets at country scale, (2) to estimate the historical and future freight volumes of waterways, and (3) to carry out the sensitivity analysis by means of the Monte Carlo approach are available at figshare [10.6084/m9.figshare.11662497].
