# Supplementary material for: Sustainability of global Golden Inland Waterways
Source: Nat Commun. 2020 Mar 25;11:1553. doi: 10.1038/s41467-020-15354-1 (PMC7096509; doi:10.1038/s41467-020-15354-1)
Supplement: Supplementary file 1 — Supplementary Information [file 41467_2020_15354_MOESM1_ESM.pdf]

Supplementary Information for

Sustainability of Global Golden Inland Waterways

Wang et al.

Supplementary Figures

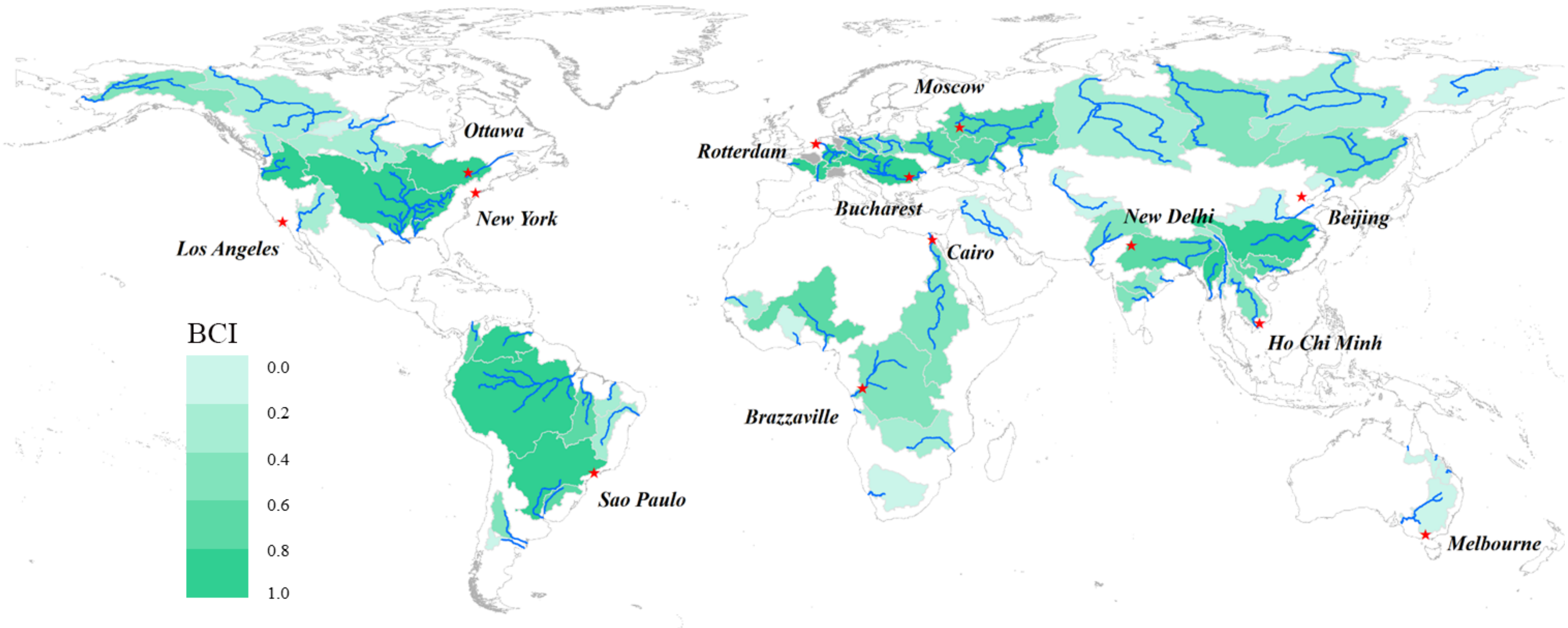

Supplementary Fig. 1 Global distribution of bearing capacity index (BCI) for large river waterways. Source data are provided as a Source Data file.

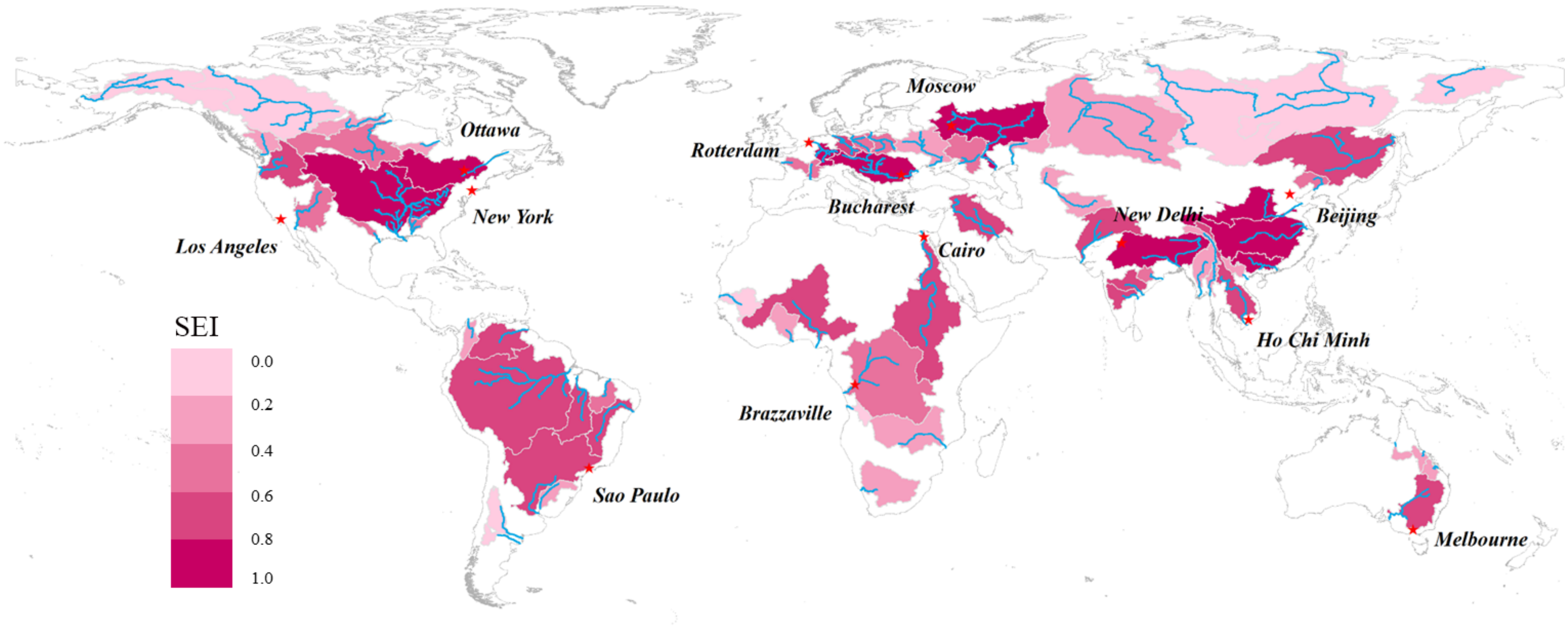

Supplementary Fig. 2 Global distribution of socio-economic index (SEI) for large river waterways. Source data are provided as a Source Data file.

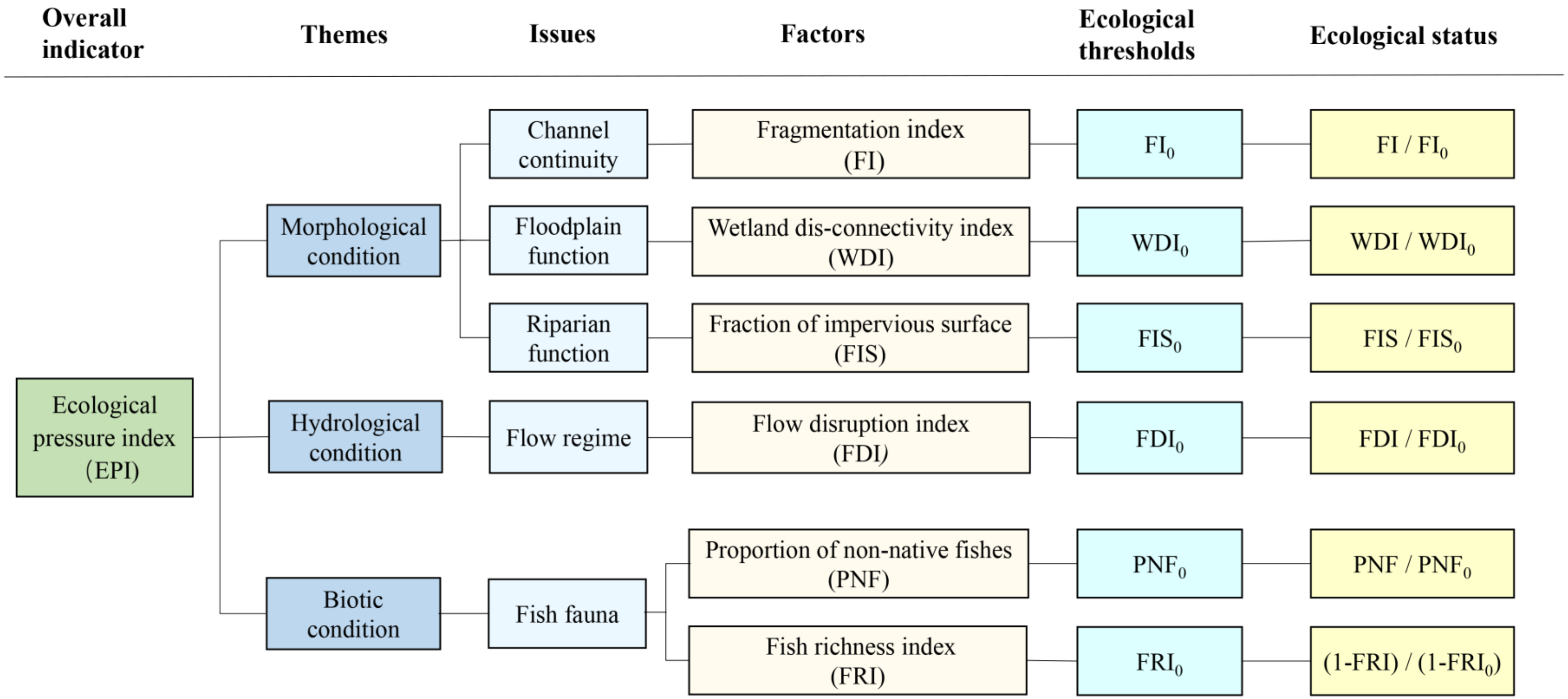

Supplementary Fig. 3 Hierarchical system for ecological pressure index (EPI) under waterway exploitation. FI_0, WDI_0, FIS_0, FDI_0, FRI_0, and PNF_0 are the critical values of FI, WDI, FIS, FDI, FRI, and PNF when waterway exploitation ratio approaching 80%, respectively.

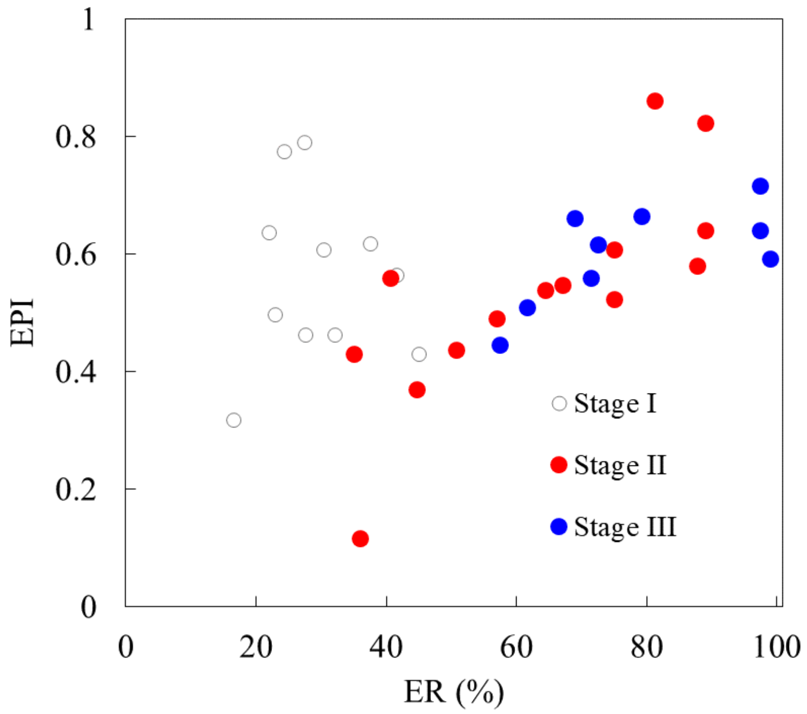

Supplementary Fig. 4 Relationship between waterway exploitation ratio (ER, %) and ecological pressure index (EPI). Development stage: I, initial; II, developing; III, developed. Source data are provided as a Source Data file.

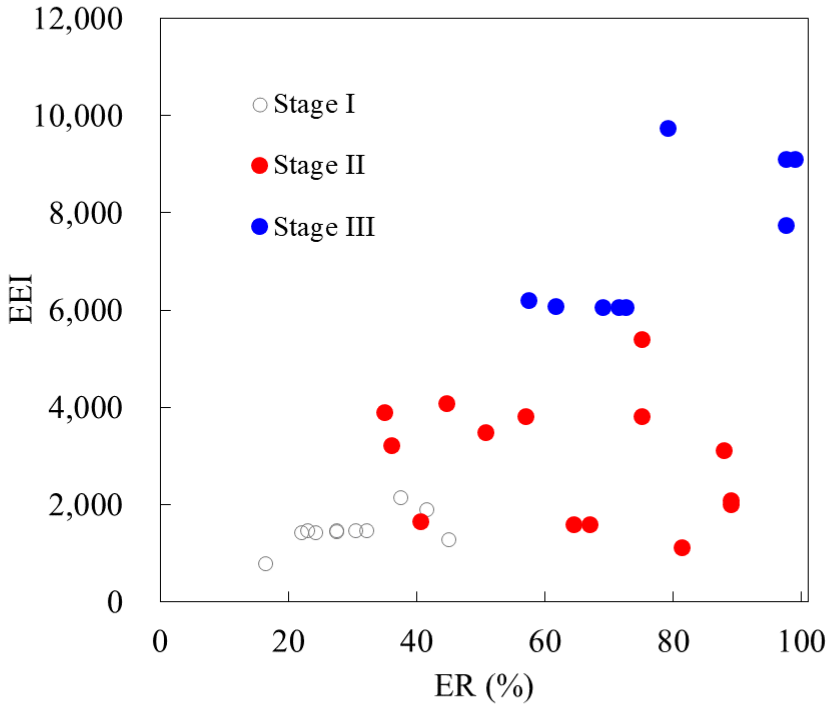

Supplementary Fig. 5 Relationship between waterway exploitation ratio (ER, %) and eco-efficiency index (EEI) for global GIWs. Development stage: I, initial; II, developing; III, developed. Source data are provided as a Source Data file.

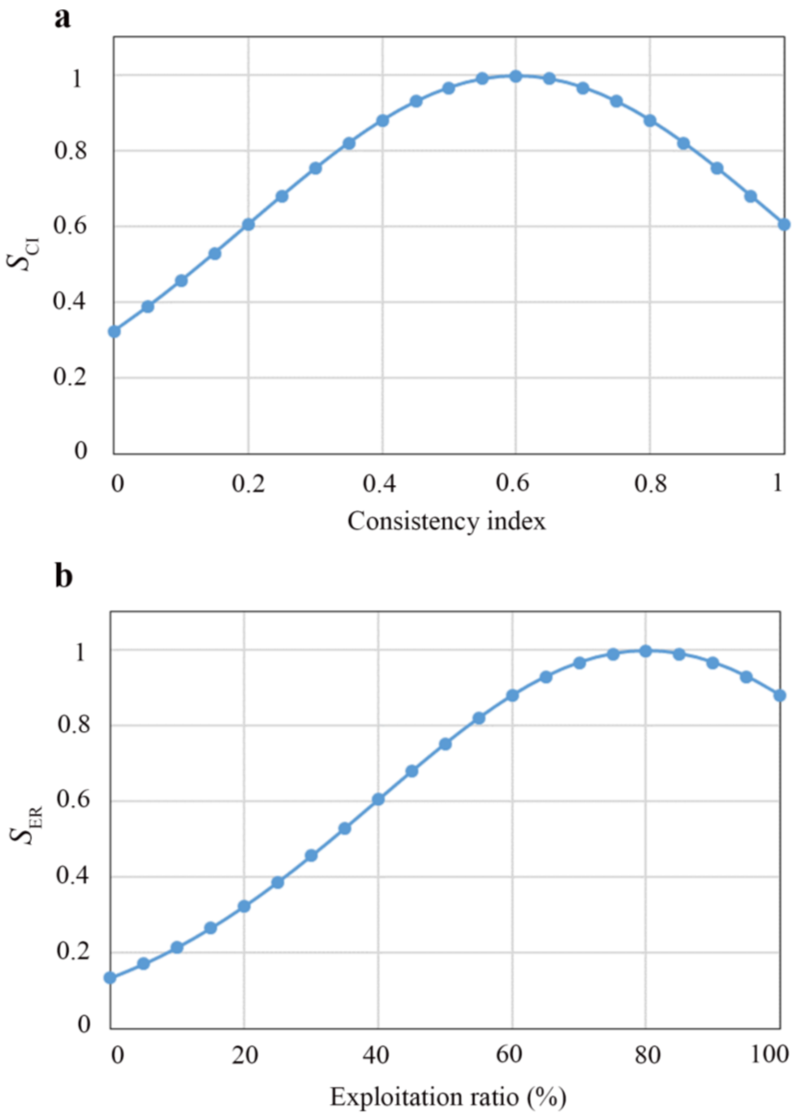

Supplementary Fig. 6 Score functions of consistency index (**a**) and exploitation ratio (**b**), used to estimate the sustainability index. S_{CI}, and S_{ER} denote the scores of a certain consistency index, and an exploitation ratio, respectively.

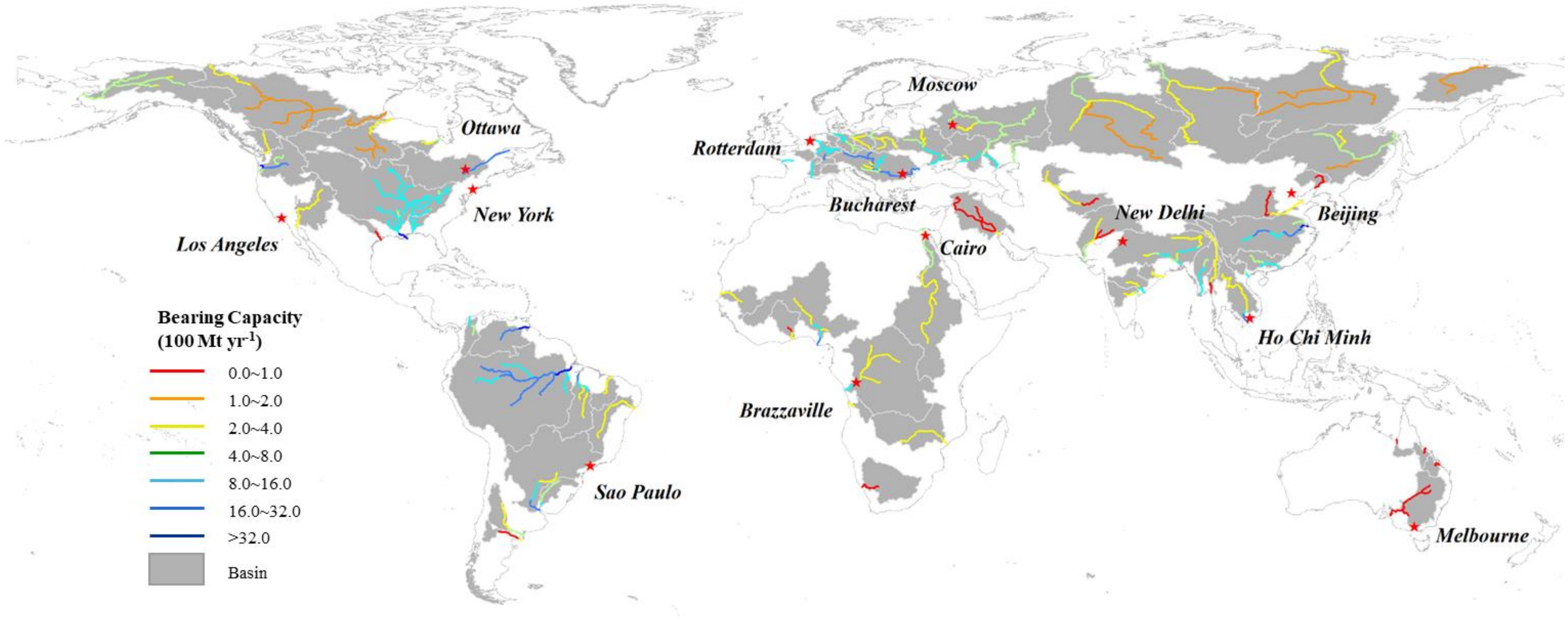

Supplementary Fig. 7 Global distribution of reach-scale bearing capacity of large river waterways. Source data are provided as a Source Data file.

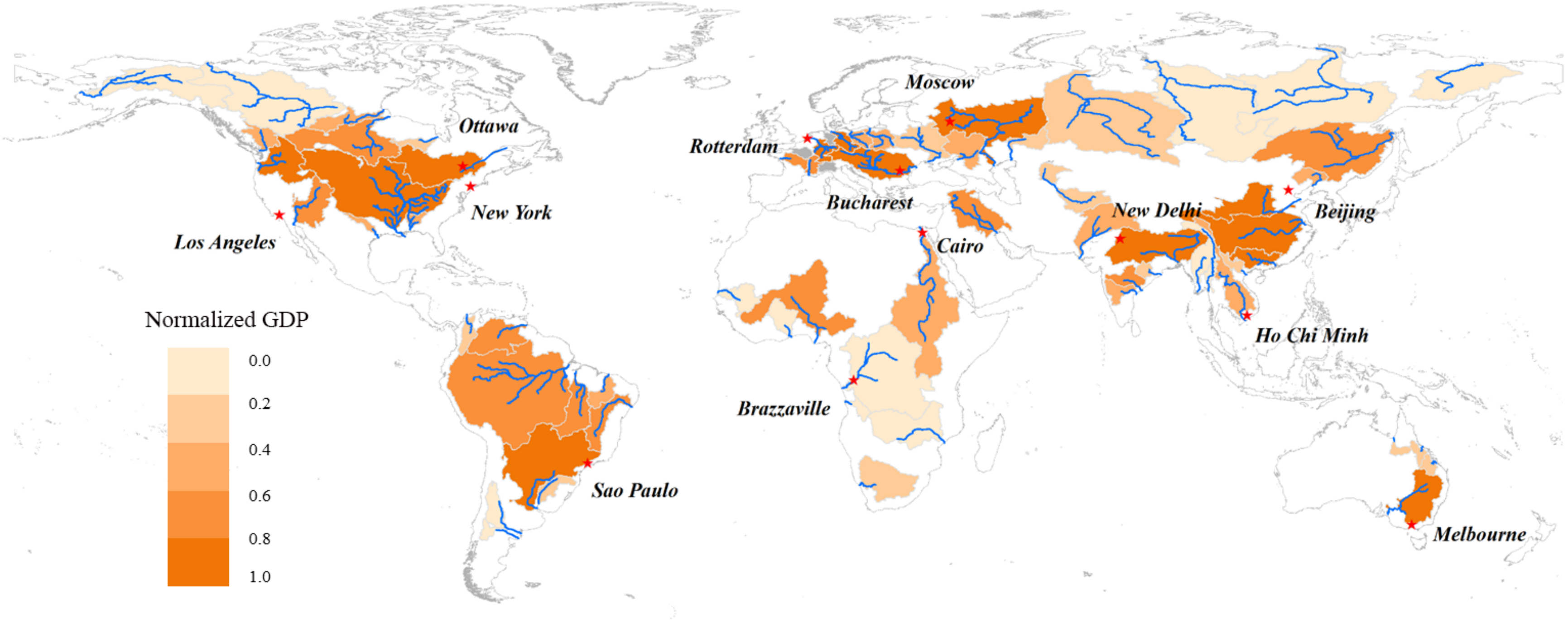

Supplementary Fig. 8 Global distribution of the normalized gross domestic product (GDP) for large river waterways. Source data are provided as a Source Data file.

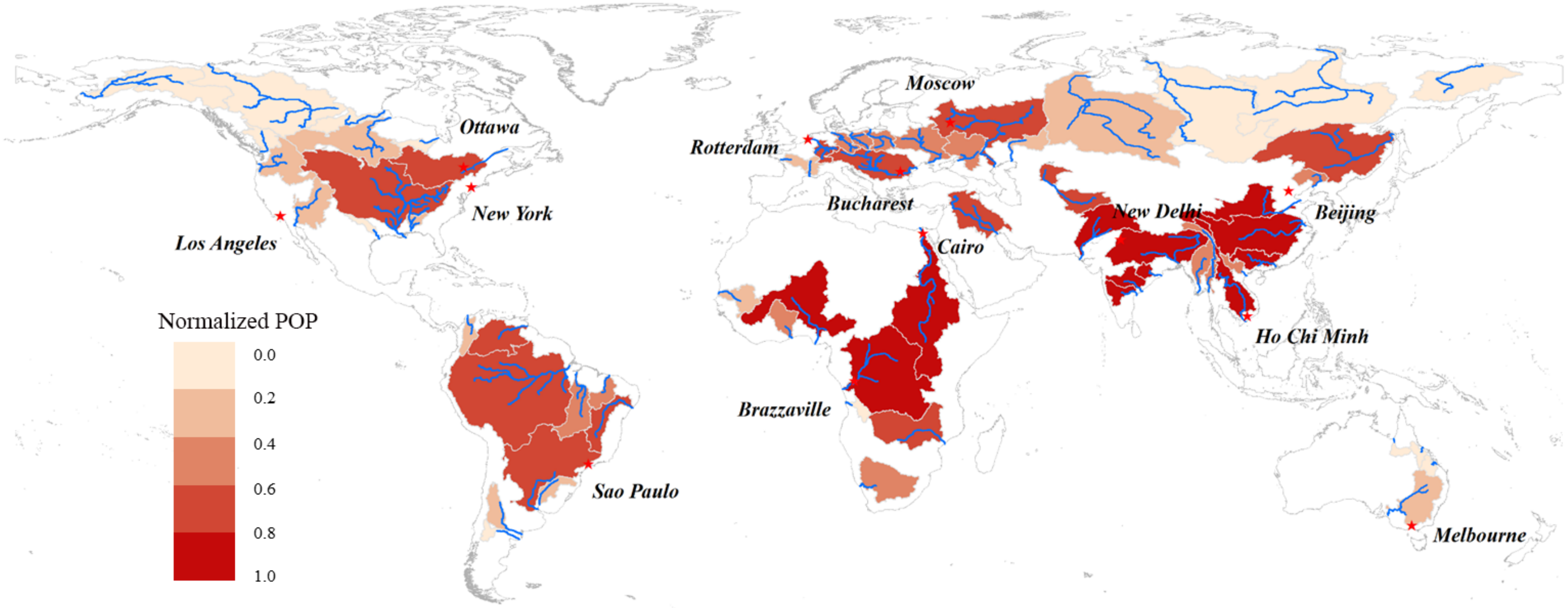

Supplementary Fig. 9 Global distribution of the normalized population (POP) for large river waterways. Source data are provided as a Source Data file.

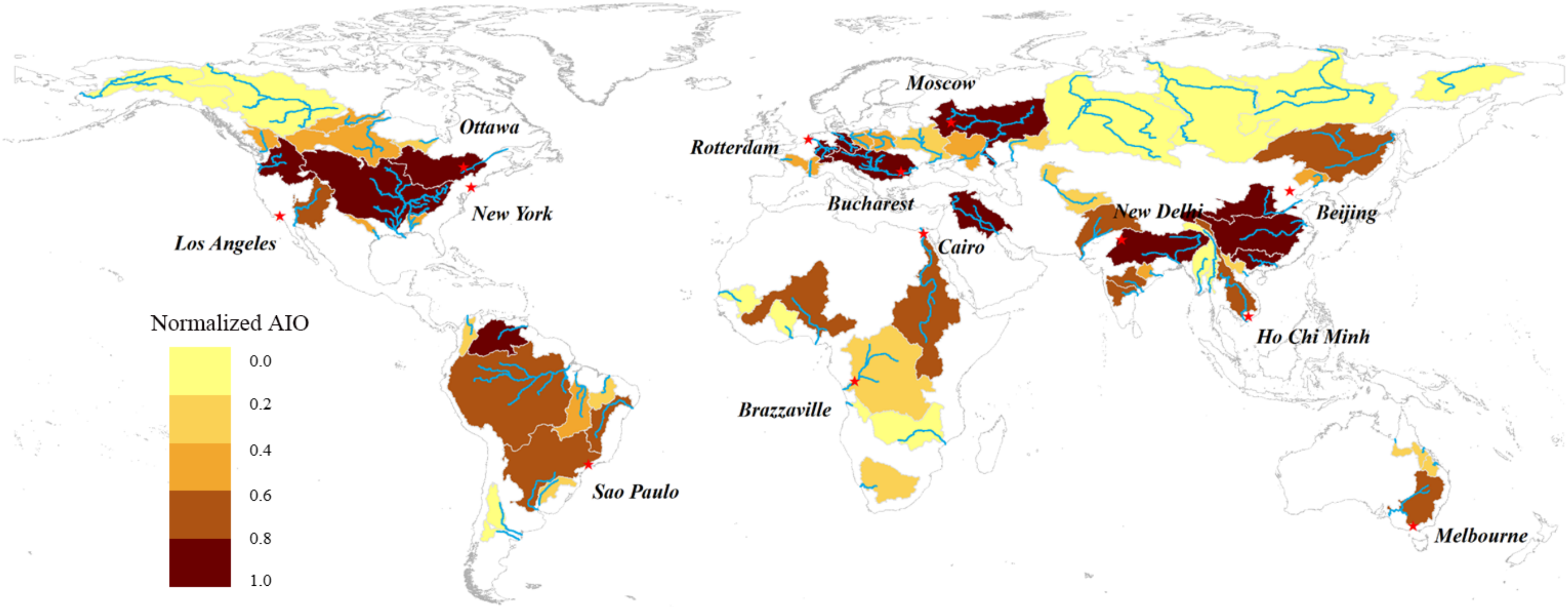

Supplementary Fig. 10 Global distribution of the normalized agriculture and industrial output (AIO) of large river waterways. Source data are provided as a Source Data file.

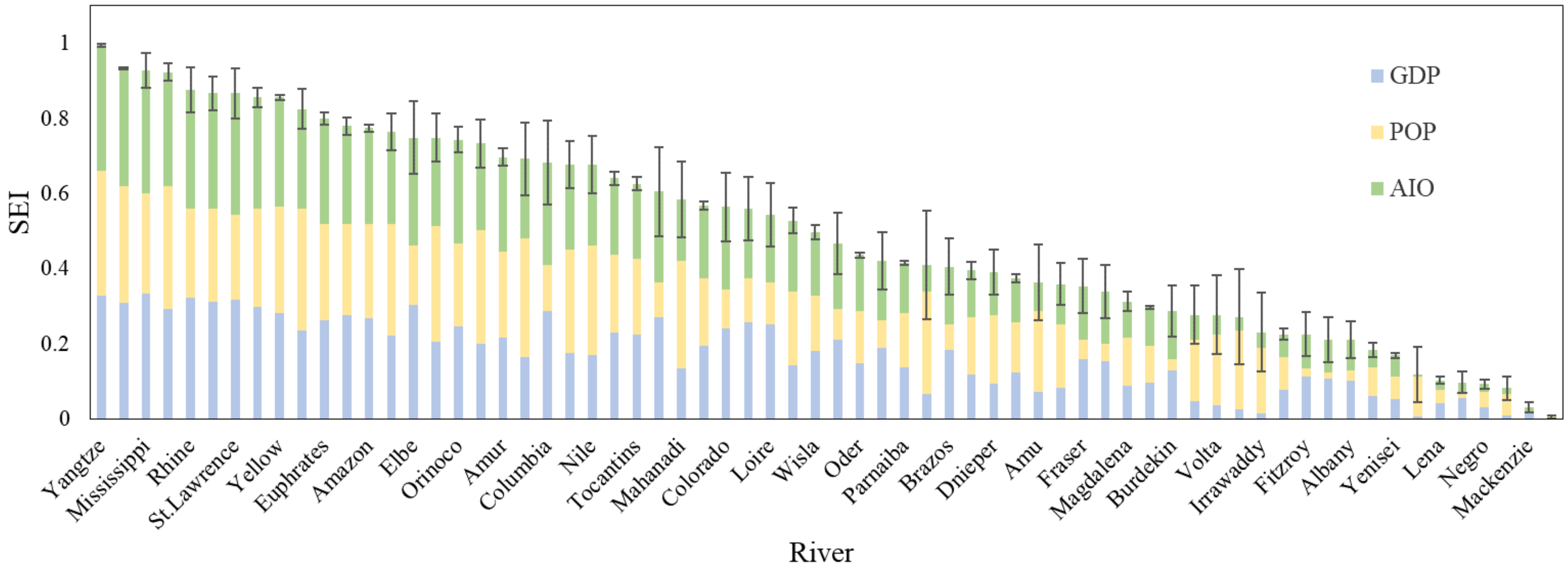

Supplementary Fig. 11 Socio-economic index (SEI) for the 66 large river inland waterways worldwide, SEI is the combination of 3 indicators with equal weightings assumed. GDP, POP, and AIO represent gross domestic product, population, and agriculture and industrial output, respectively. The error bars show the standard deviation (SD) in SEI obtained after applying the Monte Carlo approach to generate 10,000 random weights (uniformly distributed). Source data are provided as a Source Data file.

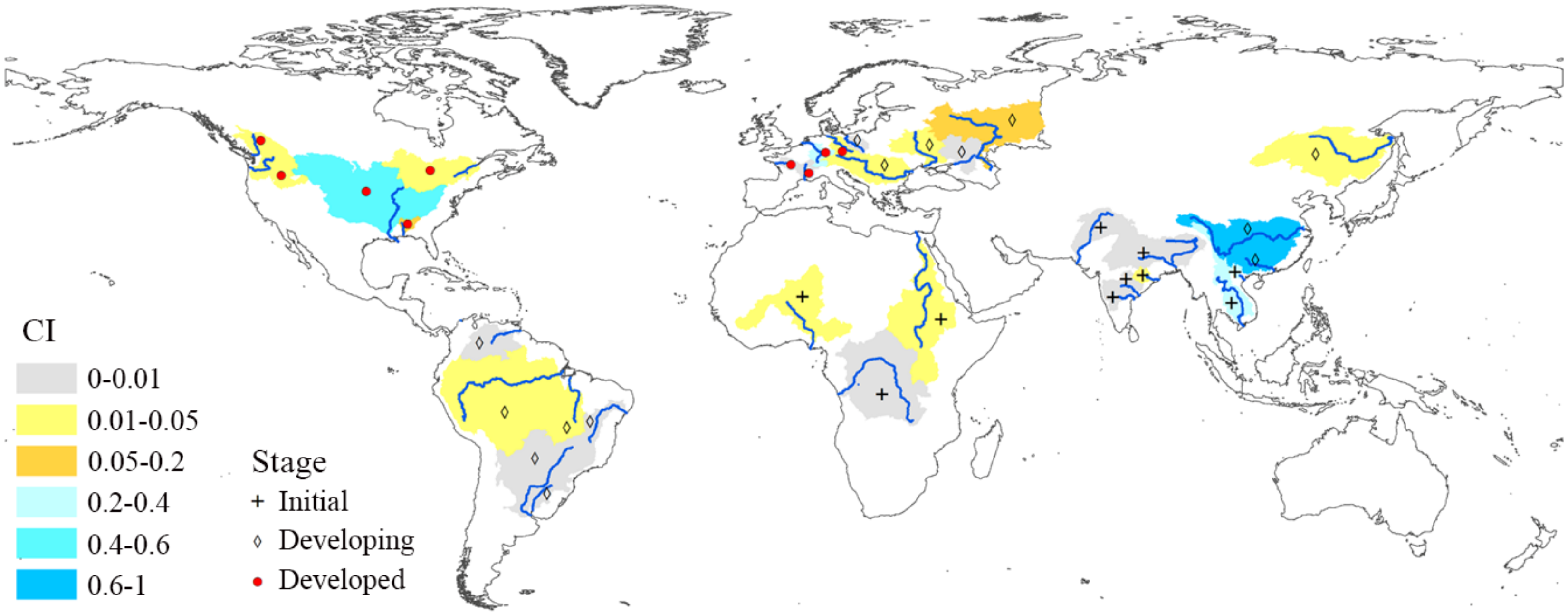

Supplementary Fig. 12 Global distribution of consistency index (CI) and corresponding development stage of golden inland waterways in 2015. Source data are provided as a Source Data file.

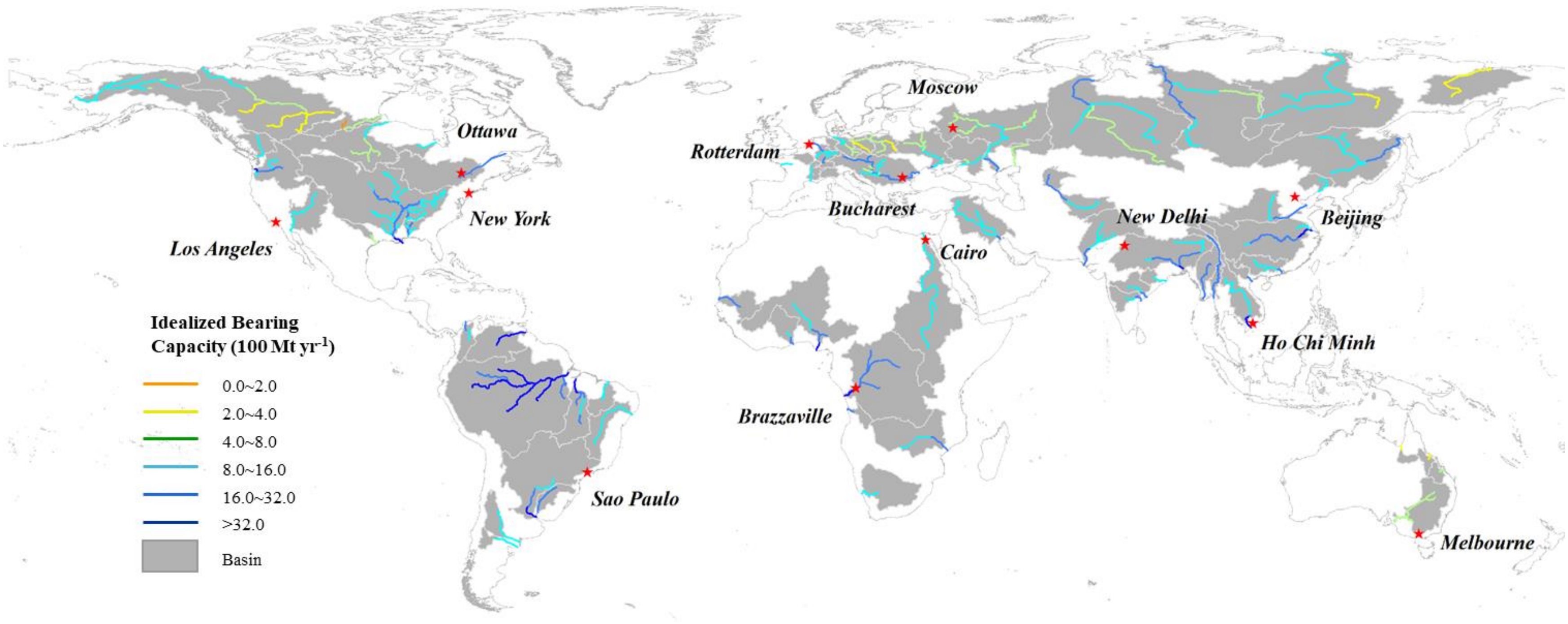

Supplementary Fig. 13 Global distribution of reach-scale idealized bearing capacity of large river waterways. Source data are provided as a Source Data file.

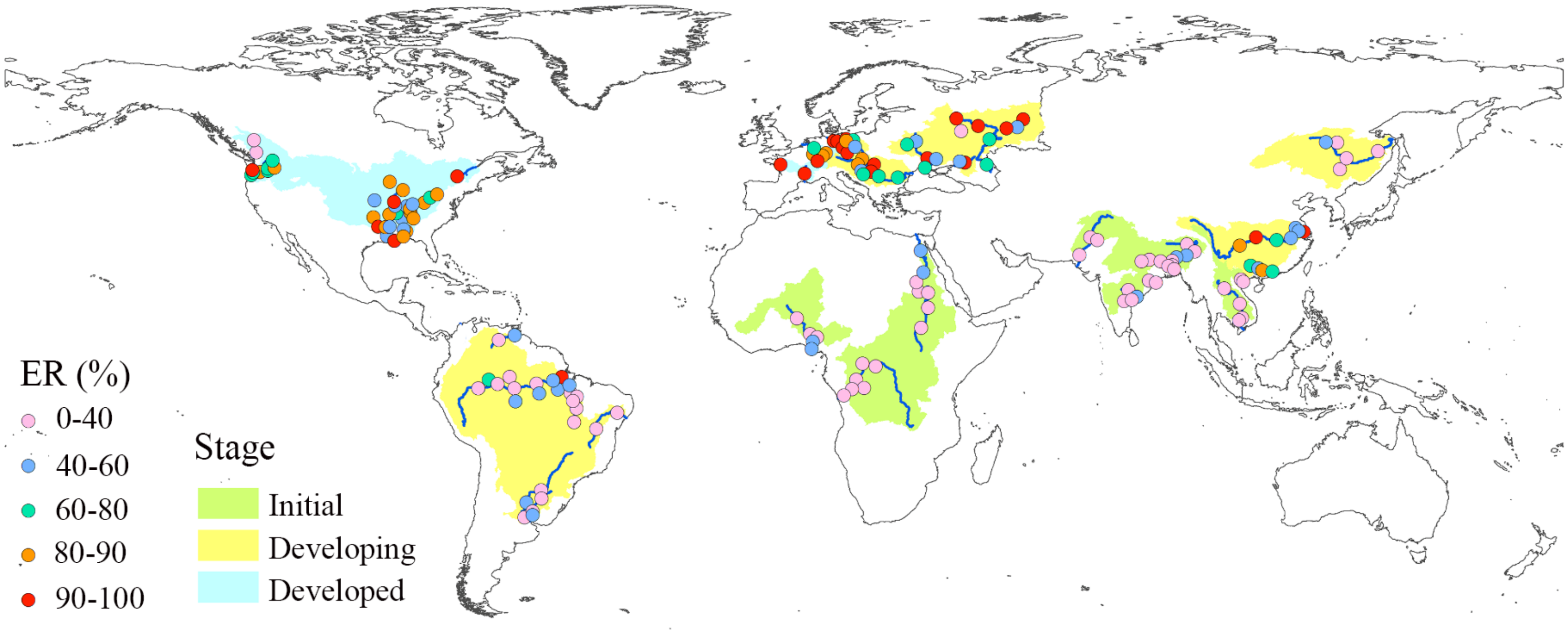

Supplementary Fig. 14 Global distribution of reach-scale exploitation ratio (ER, %) and corresponding development stage of golden inland waterways in 2015. Source data are provided as a Source Data file.

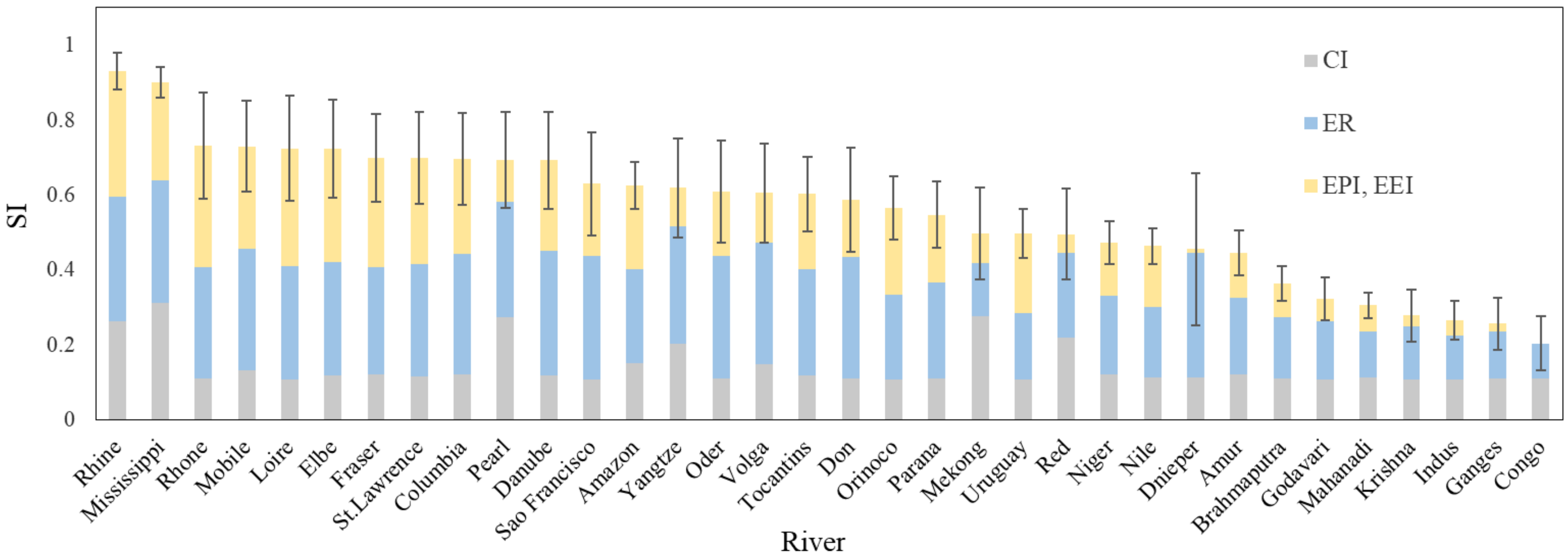

Supplementary Fig. 15 Sustainability index (SI), for 34 golden inland waterways by 2015. The error bars show the standard deviation (SD) in SI obtained after applying the Monte Carlo approach to generate 10,000 random weights (uniformly distributed). CI, ER, EPI, and EEI represent consistency index, exploitation ratio, ecological pressure index, and eco-efficiency index, respectively. Source data are provided as a Source Data file.

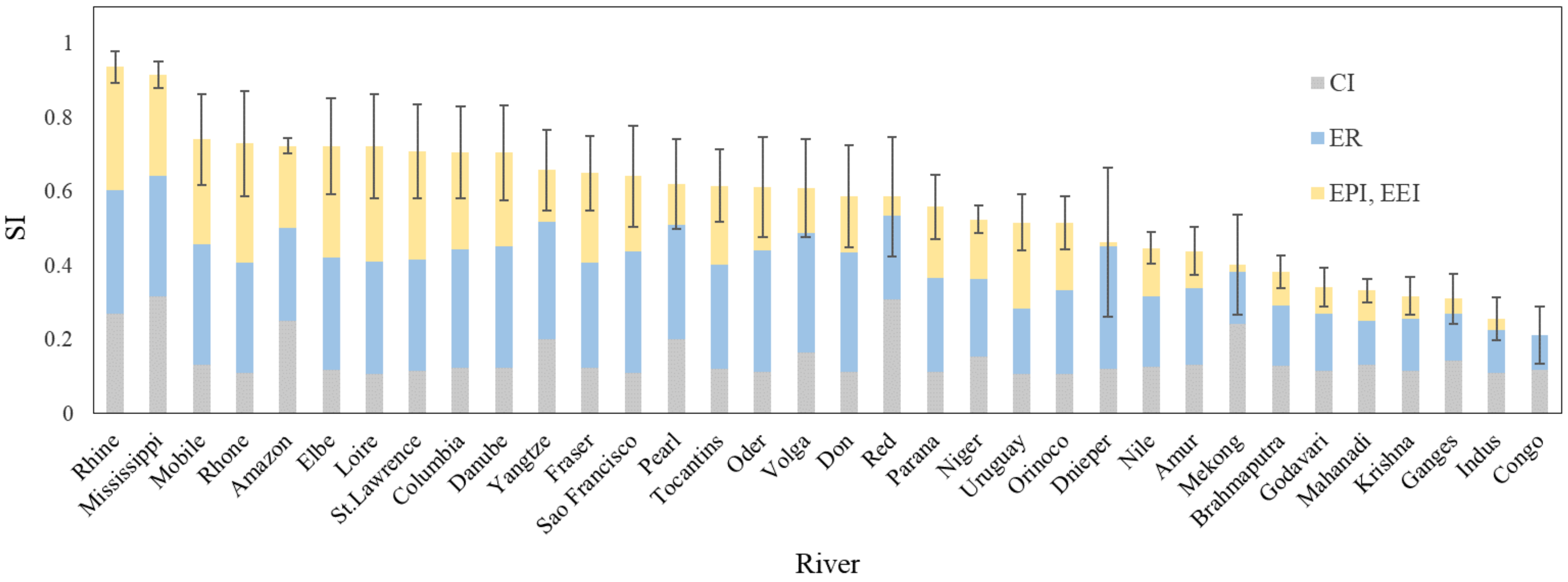

Supplementary Fig. 16 Sustainability index, SI, for 34 golden inland waterways by 2050 under the ER invariant scenario (the first scenario). The error bars show the standard deviation (SD) in SI obtained after applying the Monte Carlo approach to generate 10,000 random weights (uniformly distributed). CI, ER, EPI, and EEI represent consistency index, exploitation ratio, ecological pressure index, and eco-efficiency index, respectively. Source data are provided as a Source Data file.

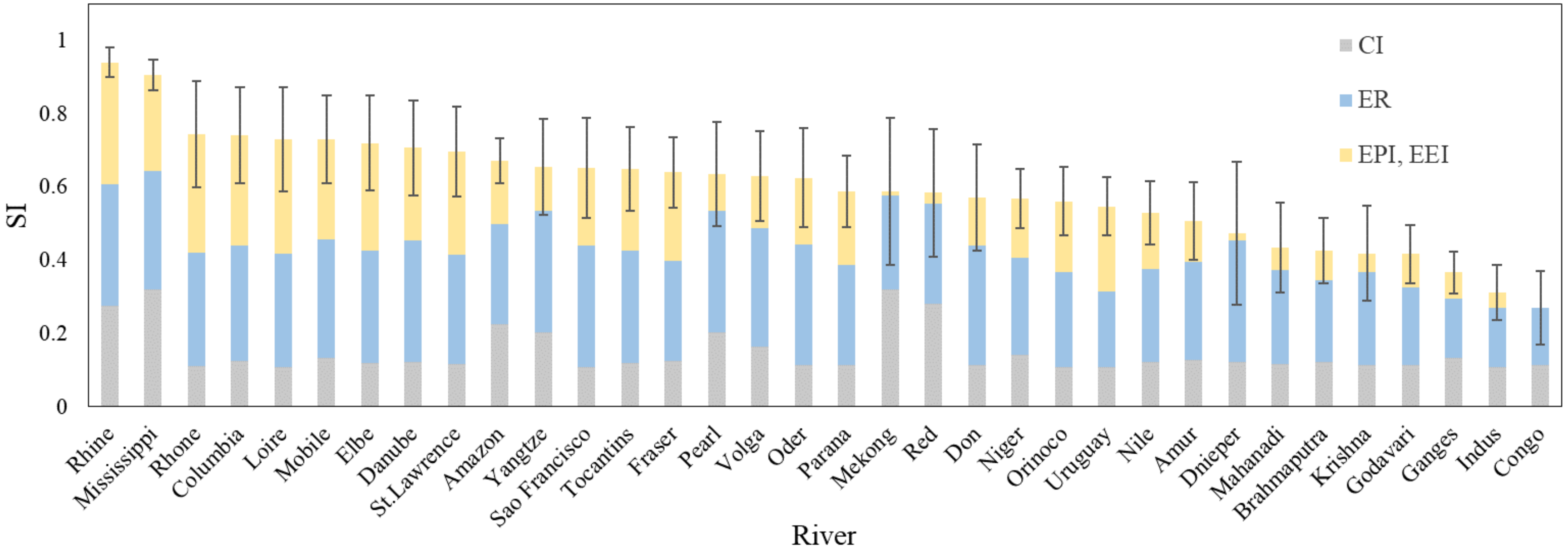

Supplementary Fig. 17 Sustainability index, SI, for 34 golden inland waterways by 2050 under the idealized scenario (the second scenario). The error bars show the standard deviation (SD) in SI obtained after applying the Monte Carlo approach to generate 10,000 random weights (uniformly distributed). CI, ER, EPI, and EEI represent consistency index, exploitation ratio, ecological pressure index, and eco-efficiency index, respectively. Source data are provided as a Source Data file.

Supplementary Tables

Supplementary Table 1 Inland waterway patterns of 66 large rivers in terms of bearing capacity index (BCI) and socio-economic index (SEI)

River	BCI	SEI	Pattern	River	BCI	SEI	Pattern
Rhine	0.86	0.88	L-L	Congo	0.57	0.41	M-M
Danube	0.92	0.87	L-L	Irrawaddy	0.77	0.23	L-S
Elbe	0.71	0.75	L-L	Magdalena	0.74	0.31	L-S
Volga	0.68	0.86	L-L	Ural	0.62	0.30	M-S
Yangtze	0.97	0.99	L-L	Ob	0.37	0.23	M-S
Pearl	0.75	0.93	L-L	Yenisei	0.45	0.17	M-S
Mississippi	0.88	0.93	L-L	Yukon	0.55	0.10	M-S
Columbia	0.94	0.68	L-L	Albany	0.43	0.21	M-S
St. Lawrence	0.95	0.87	L-L	Zambezi	0.46	0.27	M-S
Amazon	0.98	0.77	L-L	Volta	0.42	0.28	M-S
Orinoco	1.00	0.74	L-L	Yellow	0.15	0.86	S-L
Parana	0.91	0.78	L-L	Euphrates	0.12	0.80	S-L
Niger	0.69	0.76	L-L	Wisla	0.26	0.50	S-M
Rhone	0.89	0.56	L-M	Liao	0.09	0.53	S-M
Loire	0.78	0.54	L-M	Amu	0.18	0.36	S-M
Don	0.83	0.57	L-M	Murray-Darling	0.05	0.61	S-M
Red	0.72	0.36	L-M	Brazos	0.03	0.41	S-M
Mobile	0.85	0.42	L-M	Churchill	0.17	0.34	S-M
Sao Francisco	0.80	0.64	L-M	Colorado	0.34	0.56	S-M
Tocantins	0.82	0.63	L-M	Nelson	0.22	0.47	S-M
Amur	0.54	0.70	M-L	Parnaiba	0.32	0.42	S-M
Mekong	0.51	0.68	M-L	Orange	0.02	0.39	S-M
Ganges	0.63	0.92	M-L	Salween	0.23	0.28	S-S
Brahmaputra	0.58	0.83	M-L	Lena	0.25	0.10	S-S
Godavari	0.52	0.75	M-L	Kolyma	0.20	0.01	S-S
Krishna	0.48	0.73	M-L	Burdekin	0.00	0.29	S-S
Indus	0.40	0.69	M-L	Fitzroy	0.08	0.23	S-S
Nile	0.49	0.68	M-L	Flinders	0.06	0.21	S-S
Oder	0.35	0.44	M-M	Mackenzie	0.28	0.03	S-S
Dnieper	0.60	0.39	M-M	Rio Colorado	0.11	0.18	S-S
Mahanadi	0.38	0.58	M-M	Negro	0.14	0.09	S-S
Fraser	0.66	0.35	M-M	Senegal	0.31	0.12	S-S
Uruguay	0.65	0.37	M-M	Cuanza	0.29	0.08	S-S

Supplementary Table 2 Golden inland waterways (GIWs) development stages and corresponding industrialization processes and manufacturing characteristics

GIWs development stage	Economic development stage	Industrial characteristics	Added value structure (%)	GDP per capita (2010 US$)	Goods characteristics	Growth rate for IWT
initial	agricultural society	dominated by agricultural and handicraft industries	A>I	819-1638	crops	low
	early industrialization	manufacturing begins to develop, especially light industry	A > 20%, A < I	1638-3277	textiles, coals, ores, etc.	low to middle
developing	mid-industrialization	dominated by heavy and chemical industries	10% <A < 20% A < I	3277-6553	mineral building materials, oil, chemical products, steel, etc.	middle to high
	late industrialization	capital and technology-intensive industries rise up	A < 10%, I < S<50%	6553-12287	raw materials, containers, cars and other industrial products	high
	post-industrial society	capital and technology-intensive industries	A < 10%, S>50%	>12287	bulk commodities (coals, ores, etc.)	middle
developed	modernized society	Biotechnology, information and other high-tech industries develop rapidly	A < 10%, S > 70%	>25000	container transportation increase	low

A, I, and S denote agricultural, industrial, and service industries, respectively

Supplementary Table 3 Inland waterway investment and maintenance costs incurred by European countries in the period from 1995 to 2015

Year	Netherlands		France		Belgium		Serbia		Hungary		Bulgaria		Romania	
	A	B	A	B	A	B	A	B	A	B	A	B	A	B
1995	-	-	144.00	24.00	151.00	25.00	3.33	0.35	0.81	-	-	-	240.92	-
1996	-	-	79.00	26.00	125.00	27.00	3.86	1.05	0.72	-	-	-	148.28	-
1997	203.00	414.00	93.00	33.00	181.00	35.00	4.38	1.45	0.28	-	-	-	110.03	-
1998	261.00	357.00	114.00	33.00	129.00	30.00	5.92	1.75	0.33	-	-	-	107.94	-
1999	319.00	222.00	139.00	37.00	139.00	31.00	3.73	1.10	0.29	-	-	-	97.00	-
2000	402.00	362.00	783.17	46.00	152.00	48.00	4.98	1.29	0.33	-	-	-	104.84	-
2001	379.00	334.00	820.94	40.00	153.00	51.00	5.52	1.96	7.40	-	-	-	168.78	6.54
2002	512.00	441.00	950.66	41.00	153.00	51.00	13.95	4.07	5.76	-	463.27	470.45	103.69	6.08
2003	479.00	410.00	836.65	48.00	153.00	61.00	12.31	4.29	7.34	-	38.48	544.38	163.79	13.32
2004	486.00	289.00	837.60	43.00	153.00	61.00	18.66	6.13	0.75	1.01	25.60	292.85	190.50	7.65
2005	284.00	604.00	860.20	55.00	156.00	66.00	14.72	6.00	1.57	1.27	85.39	507.21	139.69	6.07
2006	312.00	377.00	978.00	61.00	162.00	67.00	29.51	7.20	3.88	24.53	196.85	619.18	213.09	17.31
2007	263.00	492.00	1052.43	58.00	178.00	76.00	23.58	11.28	4.14	33.11	405.46	787.91	358.61	28.46
2008	270.00	583.00	795.29	60.00	188.00	87.00	36.32	13.46	0.39	1.60	-	-	489.96	-
2009	361.00	693.00	693.35	61.00	188.00	131.00	19.30	10.54	3.05	0.92	-	-	536.14	-
2010	252.00	544.00	758.64	60.00	154.00	65.00	21.12	13.24	0.75	3.18	-	-	423.46	-
2011	263.00	343.00	948.95	61.00	152.00	58.00	25.76	23.03	0.21	1.57	-	-	519.04	-
2012	-	-	938.56	61.00	152.00	71.00	24.69	17.64	0.01	0.81	-	-	279.49	-
2013	-	-	744.46	61.00	167.00	66.00	15.48	16.52	0.12	0.86	-	-	268.14	-
2014	-	-	702.41	61.00	103.00	-	17.73	17.29	0.02	1.30	-	-	314.11	-
2015	-	-	700.60	59.76	291.00	-	22.32	29.82	...	1.42	-	-	505.94	-

A: inland waterway investment cost; B: inland waterway maintenance cost. Data source: https://data.oecd.org/. No data (-).

Supplementary Table 4 Development stage, bearing capacity (BC), freight transport volume (TN) of global golden inland waterways (GIWs) in 2015 and 2050

No.	GIW	2015			2050 (scenario 1)			2050 (scenario 2)		
		Development stage	BC ($Mt\ yr^{-1}$)	TN ($Mt\ yr^{-1}$)	Development stage	BC ($Mt\ yr^{-1}$)	TN ($Mt\ yr^{-1}$)	Development stage	BC ($Mt\ yr^{-1}$)	TN ($Mt\ yr^{-1}$)
1	Rhine	III	1051.78	345.82	III	1051.78	362.78	III	1022.56	362.78
2	Danube	II	1318.38	38.34	III	1318.38	47.75	III	1410.36	47.75
3	Elbe	III	756.6	18.96	III	756.6	19.66	III	741.08	19.66
4	Rhone	III	1093.95	6.36	III	1093.95	6.71	III	1049.75	6.71
5	Loire	III	862.88	1.29	III	862.88	1.36	III	845.18	1.36
6	Oder	II	230.04	2.26	III	230.04	3.63	III	224.01	3.63
7	Dnieper	II	539.02	6.5	II	539.02	17.43	II	533.7	17.43
8	Don	II	962.09	8.98	II	962.09	11.03	II	940.47	11.03
9	Volga	II	732.47	95.88	II	732.47	130.43	II	732.47	130.43
10	Yangtze	II	1735.3	2229.37	III	1735.3	4565.34	III	2046.1	4565.34
11	Pearl	II	868.82	737.48	III	868.82	1653.67	III	1061.44	1653.67
12	Amur	II	452.69	13.73	III	452.69	27.22	III	602.1	27.22
13	Red	I	774.02	198.54	II	774.02	348.6	II	1341.6	348.6
14	Mekong	I	372.876	133.37	II	372.876	342.8	II	689.01	342.8
15	Ganges	I	638.36	3.93	II	638.36	52.57	II	840.64	52.57
16	Brahmaputra	I	465.29	3.21	II	465.29	22.96	II	635.8	22.96
17	Godavari	I	431.58	0.52	II	431.58	8.9	II	594.3	8.9
18	Krishna	I	303.8	0.39	II	303.8	6.77	II	563.55	6.77
19	Mahanadi	I	254.15	4	II	254.15	13.92	II	563.55	13.92

Development stage: I, initial; II, developing; III, developed. Column No. was ranked by continents, which coincide with GIW number in Figure 2c.

Supplementary Table 4 Development stage, bearing capacity (BC), freight transport volume (TN) of global golden inland waterways (GIWs) in 2015 and 2050 (*continued*)

No.	GIW	2015			2050 (scenario 1)			2050 (scenario 2)		
		Development stage	BC (Mt yr^{-1})	TN (Mt yr^{-1})	Development stage	BC (Mt yr^{-1})	TN (Mt yr^{-1})	Development stage	BC (Mt yr^{-1})	TN (Mt yr^{-1})
20	Indus	I	269.06	0.5	II	269.06	1.38	II	391.36	1.38
21	Mississippi	II	1093.3	497.28	III	1093.3	523.51	III	1063.14	523.51
22	Columbia	III	1395.18	49.48	III	1395.18	52.13	III	1354.74	52.13
23	Mobile	III	982.41	55.68	III	982.41	58.66	III	968.67	58.66
24	St. Lawrence	III	1719.87	36.25	III	1719.87	38.21	III	1703.12	38.21
25	Fraser	III	733.13	26.15	III	733.13	27.61	III	701.25	27.61
26	Amazon	II	2039.04	51.92	II	2039.04	111.07	II	2152.32	111.07
27	Orinoco	II	2624.78	3.69	II	2624.78	5.11	II	3053.44	5.11
28	Parana	II	1187.9	10.38	II	1187.9	15.06	II	1288.65	15.06
29	Sao Francisco	II	933.75	0.08	II	933.75	3.92	II	964.88	3.92
30	Tocantins	II	934.23	25.26	II	934.23	32.08	II	1048.96	32.08
31	Uruguay	II	663.95	0.44	II	663.95	0.7	II	777.77	0.7
32	Congo	I	470.25	1.67	I	470.25	12.16	I	883.5	12.16
33	Nile	I	358.7	3.71	II	358.7	15.91	II	486.54	15.91
34	Niger	I	751.71	24.26	II	751.71	77.24	II	957.71	77.24

Development stage: I, initial; II, developing; III, developed. Column No. was ranked by continents, which coincide with GIW number in Figure 2c.

Supplementary Table 5 Consistency index (CI), exploitation ratio (ER), ecological pressure index (EPI), and eco-efficiency index (EEI) of global golden inland waterways (GIWs) in 2015 and two scenarios in 2050

No.	GIW	2015				2050 (scenario 1)				2050 (scenario 2)			
		CI	ER	EPI	EEI	CI	ER	EPI	EEI	CI	ER	EPI	EEI
1	Rhine	0.33	0.79	0.66	9755.51	0.34	0.79	0.66	14320.34	0.35	0.77	0.60	14320.34
2	Danube	0.03	0.75	0.60	5399.23	0.04	0.75	0.60	10154.84	0.03	0.79	0.61	10154.84
3	Elbe	0.03	0.98	0.64	7758.53	0.03	0.98	0.64	11661.64	0.03	0.96	0.70	11661.64
4	Rhone	0.01	0.99	0.59	9117.38	0.01	0.99	0.59	13129.87	0.01	0.95	0.70	13129.87
5	Loire	0.00	0.98	0.71	9117.38	0.00	0.98	0.71	13129.87	0.00	0.96	0.70	13129.87
6	Oder	0.01	0.88	0.57	3121.74	0.02	0.88	0.57	6125.25	0.02	0.86	0.65	6125.25
7	Dnieper	0.01	0.81	0.83	1121.80	0.03	0.81	0.83	2456.84	0.03	0.81	0.62	2456.84
8	Don	0.01	0.89	0.63	2018.18	0.01	0.89	0.63	3975.41	0.01	0.87	0.66	3975.41
9	Volga	0.09	0.89	0.79	2102.62	0.13	0.89	0.79	4096.02	0.13	0.89	0.67	4096.02
10	Yangtze	1.00	0.67	0.55	1595.50	1.00	0.67	0.55	3668.57	1.00	0.79	0.61	3668.57
11	Pearl	0.85	0.65	0.53	1595.50	1.00	0.65	0.53	3436.51	1.00	0.79	0.61	3436.51
12	Amur	0.03	0.41	0.56	1653.08	0.06	0.41	0.56	3262.04	0.05	0.54	0.47	3262.04
13	Red	0.23	0.45	0.43	1271.44	0.45	0.45	0.43	2313.62	0.37	0.55	0.47	2313.62
14	Mekong	0.36	0.28	0.46	1446.55	0.92	0.28	0.46	2154.40	0.50	0.51	0.45	2154.40
15	Ganges	0.01	0.24	0.77	1423.25	0.08	0.24	0.77	2756.32	0.06	0.32	0.34	2756.32
16	Brahmaputra	0.01	0.32	0.46	1466.76	0.05	0.32	0.46	2910.42	0.04	0.44	0.41	2910.42
17	Godavari	0.00	0.31	0.61	1463.30	0.02	0.31	0.61	2900.58	0.01	0.42	0.40	2900.58
18	Krishna	0.00	0.28	0.79	1463.30	0.02	0.28	0.79	2900.58	0.01	0.51	0.45	2900.58
19	Mahanadi	0.02	0.23	0.50	1463.30	0.05	0.23	0.50	2900.58	0.02	0.51	0.45	2900.58
20	Indus	0.00	0.22	0.64	1417.91	0.01	0.22	0.64	2499.39	0.00	0.32	0.34	2499.39

Development stage: I, initial; II, developing; III, developed. Column GIW No. was ranked by continents, which coincide with GIW number in Figure 2c.

Supplementary Table 5 Consistency index (CI), exploitation ratio (ER), ecological pressure index (EPI), and eco-efficiency index (EEI) of global golden inland waterways (GIWs) in 2015 and two scenarios in 2050 (*continued*)

No.	GIW	2015				2050 (scenario 1)				2050 (scenario 2)			
		CI	ER	EPI	EEI	CI	ER	EPI	EEI	CI	ER	EPI	EEI
21	Mississippi	0.45	0.73	0.61	6064.99	0.48	0.73	0.61	10774.22	0.49	0.71	0.56	10774.22
22	Columbia	0.04	0.69	0.66	6064.99	0.04	0.69	0.66	10774.22	0.04	0.67	0.54	10774.22
23	Mobile	0.06	0.72	0.55	6064.99	0.06	0.72	0.55	10774.22	0.06	0.71	0.56	10774.22
24	St. Lawrence	0.02	0.62	0.51	6079.25	0.02	0.62	0.51	10576.07	0.02	0.61	0.51	10576.07
25	Fraser	0.04	0.58	0.45	6212.19	0.04	0.58	0.45	8429.14	0.04	0.55	0.47	8429.14
26	Amazon	0.03	0.36	0.12	3215.17	0.05	0.36	0.12	5325.00	0.05	0.40	0.39	5325.00
27	Orinoco	0.00	0.45	0.37	4098.61	0.00	0.45	0.37	5622.28	0.00	0.52	0.46	5622.28
28	Parana	0.01	0.51	0.43	3486.01	0.01	0.51	0.43	6103.49	0.01	0.55	0.47	6103.49
29	Sao Francisco	0.00	0.75	0.52	3820.66	0.00	0.75	0.52	6596.82	0.00	0.78	0.60	6596.82
30	Tocantins	0.03	0.57	0.49	3820.66	0.03	0.57	0.49	6596.82	0.03	0.64	0.53	6596.82
31	Uruguay	0.00	0.35	0.43	3899.08	0.00	0.35	0.43	7537.73	0.00	0.41	0.40	7537.73
32	Congo	0.00	0.17	0.32	781.50	0.03	0.17	0.32	1499.69	0.01	0.31	0.34	1499.69
33	Nile	0.01	0.38	0.61	2146.01	0.04	0.38	0.61	3765.50	0.03	0.51	0.45	3765.50
34	Niger	0.03	0.42	0.56	1897.65	0.10	0.42	0.56	4004.21	0.08	0.53	0.46	4004.21

Development stage: I, initial; II, developing; III, developed. Column No. was ranked by continents, which coincide with GIW number in Figure 2c.

Supplementary Table 6 Navigation standard of inland waterways grade of China[1], and the corresponding average tonnage (M), minimum waterway maintenance depth, the longitudinal domain lengths of upstream (l_u) and downstream ships (l_d), and bearing capacity (BC) [2,3]

Waterway grade	M (t)	Minimum waterway maintenance depth (m)	l_u	l_d	BC (10^8 t yr^{-1})
I-1	50000	12.5	2.6	4.5	86.85
I-2	20000	9.0-12.5	3.3	5.2	45.35
I-3	10000	6.0-9.0	2.7	4.3	26.45
I-4	7000	4.5-6.0	-	-	20.95
I-5	5000	4.0-4.5	2.8	4.8	17.20
I-6	3000	3.5-4.0	3.5	5.2	11.05
II	2000	2.6-3.5	2.9	4.5	8.85
III	1000	2.0-2.6	3.7	5.0	5.65
IV	500	1.6-2.0	3.2	4.6	3.03
V	300	1.4-1.6	-	-	2.24
VI	100	1.0-1.4	-	-	0.86
VII	50	0.7-1.0	-	-	0.58

Supplementary Table 7 Minimum maintenance depth of inland waterways

River	Minimum maintenance depth (m)	River	Minimum maintenance depth (m)
Rhine	2.6-3.5	Fitzroy	1.1
Danube	1.4-4.5	Flinders	1.0
Elbe	2.6-3.0	Murray-Darling	1.3
Rhone	3.5-3.5	Mississippi	2.1-13.7
Loire	2.6-2.6	Columbia	2.1-13.4
Oder	1.6-2.5	Mobile	2.7-12.2
Wisla	1.4-3.5	St. Lawrence	2.4-9.1
Dnieper	1.6-4.5	Yukon	2.1-3.7
Don	2.0-4.0	Albany	1.4-1.8
Volga	1.6-4.5	Brazos	1.2
Ural	3.0-3.0	Churchill	1.4-1.6
Yangtze	2.0-12.6	Colorado	1.4
Pearl	2.0-4.5	Fraser	2.0-2.6
Amur	1.8-3.5	Nelson	1.5-2.0
Liao	1.2	Mackenzie	1.8-3.7
Yellow	1.2-1.4	Amazon	3.6-18.0
Red	2.4-3.4	Rio Colorado	1.1-1.2
Mekong	1.4-4.5	Magdalena	2.2-3.5
Salween	1.1-1.4	Negro	1.2-1.4
Irrawaddy	2.6-2.9	Orinoco	4.0-10.5
Ganges	1.4-3.8	Parana	1.6-6.0
Brahmaputra	1.6-2.6	Parnaiba	1.5
Godavari	1.4-2.6	Sao Francisco	1.8-4.0
Krishna	1.6-1.8	Tocantins	1.8-4.5
Mahanadi	1.4-1.6	Uruguay	2.0-3.5
Indus	1.2-2.0	Congo	1.6-3.5
Amu	1.1-1.5	Nile	1.4-2.3
Euphrates	0.8-1.6	Niger	1.4-6.0
Ob	1.6-3.6	Orange	1.2
Yenisei	1.8-3.7	Zambezi	1.6
Lena	1.8-2.6	Senegal	1.4
Kolyma	2.0	Cuanza	1.5
Burdekin	1.1	Volta	1.5-1.7

Supplementary Table 8 Government agencies managing 66 large inland waterways worldwide

River	Data source
Rhine	http://www.unece.org/trans/resources/publications/
Danube	http://www.unece.org/trans/resources/publications/
Elbe	http://www.unece.org/trans/resources/publications/
Rhone	http://www.unece.org/trans/resources/publications/
Loire	http://www.unece.org/trans/resources/publications/
Oder	http://www.unece.org/trans/resources/publications/
Wisla	http://www.unece.org/trans/resources/publications/
Dnieper	http://www.unece.org/trans/resources/publications/
Don	http://www.morflot.ru/deyatelnost/vvt.html
Volga	http://www.morflot.ru/deyatelnost/vvt.html
Ural	http://www.morflot.ru/deyatelnost/vvt.html
Yangtze	http://www.cjhdj.com.cn/
Pearl	http://www.zjhw.gov.cn/
Amur	http://www.mot.gov.cn/
Liao	http://www.mot.gov.cn/
Yellow	http://www.mot.gov.cn/
Red	http://en.viwa.gov.vn/home
Mekong	http://en.viwa.gov.vn/home
Salween	http://www.iwt.gov.mm/en
Irrawaddy	http://www.iwt.gov.mm/en
Ganges	http://www.iwai.nic.in/
Brahmaputra	http://www.iwai.nic.in/
Godavari	http://www.iwai.nic.in/
Krishna	http://www.iwai.nic.in/
Mahanadi	http://www.iwai.nic.in/
Indus	http://pakirsa.gov.pk/
Amu	http://www.cawater-info.net/bd/index_e.htm
Euphrates	http://www.fao.org/nr/water/aquastat/basins/euphrates-tigris
Ob	http://www.morflot.ru/deyatelnost/vvt.html
Yenisei	http://www.morflot.ru/deyatelnost/vvt.html
Lena	http://www.morflot.ru/deyatelnost/vvt.html
Kolyma	http://www.morflot.ru/deyatelnost/vvt.html
Burdekin	http://www.bom.gov.au/
Fitzroy	http://www.bom.gov.au/
Flinders	http://www.bom.gov.au/
Murray-Darling	https://www.mdba.gov.au/

Supplementary Table 8 Government agencies managing 66 large inland waterways worldwide (*continued*)

River	Data source
Mississippi	http://www.navigationdatacenter.us
Columbia	http://www.navigationdatacenter.us
Mobile	http://www.navigationdatacenter.us
St. Lawrence	http://www.greatlakes-seaway.com
Yukon	http://www.navigationdatacenter.us
Albany	https://open.canada.ca/en
Brazos	https://open.canada.ca/en
Churchill	https://open.canada.ca/en
Colorado	https://open.canada.ca/en
Fraser	https://open.canada.ca/en
Nelson	https://open.canada.ca/en
Mackenzie	https://open.canada.ca/en
Amazon	http://infraestrutura.gov.br/index.php
Rio Colorado	https://www.argentina.gob.ar
Magdalena	https://colaboracion.dnp.gov.co
Negro	https://www.argentina.gob.ar
Orinoco	http://www.mtc.gob.ve
Parana	https://www.argentina.gob.ar
Parnaiba	http://infraestrutura.gov.br/index.php
Sao Francisco	http://infraestrutura.gov.br/index.php
Tocantins	http://infraestrutura.gov.br/index.php
Uruguay	http://www.mtop.gub.uy/
Congo	http://www.cicos.info/siteweb/
Nile	http://mot.gov.eg/
Niger	http://www.abn.ne/
Orange	https://www.dwa.gov.za/orange/default.aspx
Zambezi	http://www.zambezira.org/
Senegal	https://www.nap.edu/read/10546/chapter/3
Cuanza	http://www.mintrans.gov.ao/
Volta	https://www.ghanamaritime.org/index.php

Supplementary Table 9 Freeze duration of large rivers

River	Freeze duration (day)
Lena	240
Kolyma	240
Mackenzie	240
Ob	210
Yenisei	210
Yukon	210
Wisla	150
Amur	150
Churchill	150
Nelson	150
St. Lawrence	140
Oder	120
Volga	120
Ural	120
Dnieper	90
Don	90
Elbe	45
Fraser	30

Supplementary Table 10 Number of golden inland waterways (GIWs) identified by varying the threshold values

BCI_T	SEI_T	Number of GIWs	Relative percentage to baseline case
0.50	0.33	28	-18%
0.17	0.33	38	12%
0.33	0.55	27	-21%
0.33	0.17	41	21%

BCI_T and SEI_T are threshold values of bearing capacity index (BCI) and socio-economic index (SEI) to identify the GIWs.

Supplementary Table 11 Data sources for freight transport volume of golden inland waterways (GIWs)

No.	River	Data source
1	Rhine	https://ec.europa.eu/eurostat/web/products-datasets/-/ttr00007
2	Danube	https://ec.europa.eu/eurostat/web/products-datasets/-/ttr00007
3	Elbe	https://ec.europa.eu/eurostat/web/products-datasets/-/ttr00007
4	Rhone	https://ec.europa.eu/eurostat/web/products-datasets/-/ttr00007
5	Loire	https://ec.europa.eu/eurostat/web/products-datasets/-/ttr00007
6	Oder	https://ec.europa.eu/eurostat/web/products-datasets/-/ttr00007
7	Dnieper	https://mtu.gov.ua/files/USACEreport.pdf
8	Don	https://www.fedstat.ru/
9	Volga	https://www.fedstat.ru/
10	Yangtze	www.cjhdj.com.cn
11	Pearl	https://zjhy.mot.gov.cn/
12	Amur	http://www.mot.gov.cn/tongjishuju/shuiyun/
13	Red	https://openknowledge.worldbank.org/bitstream/handle/10986/16321/9781464801051.pdf?sequence=1&isAllowed=y
14	Mekong	https://openknowledge.worldbank.org/bitstream/handle/10986/16321/9781464801051.pdf?sequence=1&isAllowed=y
15	Ganges	http://www.manthan-india.org/wp-content/uploads/2018/04/Strategic-Status-Report-on-Inland-Waterways-V5-26-Apr-17-FINAL.pdf
16	Brahmaputra	http://www.manthan-india.org/wp-content/uploads/2018/04/Strategic-Status-Report-on-Inland-Waterways-V5-26-Apr-17-FINAL.pdf
17	Godavari	http://iwai.nic.in/WriteReadData/l892s/file112-54022272.pdf

Column No. was ranked by continents, which coincide with GIW number in Figure 2c.

Supplementary Table 11 Data sources for freight transport volume of golden inland waterways (GIWs) (*continued*)

No.	River	Data sources
18	Krishna	http://iwai.nic.in/WriteReadData/l892s/file112-54022272.pdf
19	Mahanadi	http://iwai.nic.in/showfile.php?lid=939
20	Indus	https://iwt.punjab.gov.pk/
21	Mississippi	https://www.iwr.usace.army.mil/About/Technical-Centers/WCSC-Waterborne-Commerce-Statistics-Center/
22	Columbia	https://www.iwr.usace.army.mil/About/Technical-Centers/WCSC-Waterborne-Commerce-Statistics-Center/
23	Mobile	https://www.iwr.usace.army.mil/About/Technical-Centers/WCSC-Waterborne-Commerce-Statistics-Center/
24	St. Lawrence	http://www.greatlakes-seaway.com/en/seaway/index.html
25	Fraser	http://faculty.bcitbusiness.org/kevinw/documents/Fraser_River_SSS_Final.pdf
26	Amazon	http://www.transportes.gov.br/images/TRANSPORTE_HIDROVIARIO/PHE/PlanReport.pdf
27	Orinoco	www.mtc.gob.ve
28	Parana	http://www.transportes.gov.br/images/TRANSPORTE_HIDROVIARIO/PHE/PlanReport.pdf
29	Sao Francisco	http://www.transportes.gov.br/images/TRANSPORTE_HIDROVIARIO/PHE/PlanReport.pdf
30	Tocantins	http://www.transportes.gov.br/images/TRANSPORTE_HIDROVIARIO/PHE/PlanReport.pdf
31	Uruguay	http://www.mtop.gub.uy/
32	Congo	http://www.cicos.info/siteweb/
33	Nile	https://www.wwinn.org/nile
34	Niger	https://nigerianports.gov.ng/ports-statistics/

Column No. was ranked by continents, which coincide with GIW number in Figure 2c.

Supplementary Table 12 Elastic coefficient estimated for golden inland waterways at different development stages and gross domestic product (GDP) per capita

Development stage	GDP per capita (US$)	Elastic coefficient
developed	1000-4000	0.4-0.6
	4000-7000	0.6-0.9
	7000-11000	0.9-1.3
	11000-20000	0.7-0.9
	20000-25000	0.4-0.7
	> 25000	0.4-1
developing	1000-2000	1.3-2.0
	2000-7000	0.7-1.3
	> 7000	< 0.5

Supplementary Table 13 Interpretation and data source for metrics in the Methods

Metric	Interpretation	Method and Data source
BC	Bearing capacity of inland waterways which is approximated by the theoretical annual freight volume that can pass through a given waterway cross-section	Equation (1)
M	Average tonnage of inland vessels estimated based on waterway grade determined by minimum waterway maintenance depth	Supplementary Table 6
T	Number of navigable days per year estimated using data on freeze-up duration	Supplementary Table 9
q_{h}	Hourly basic inland waterway traffic capacity	Equation (2)
l_{u}	Longitudinal domain lengths of upstream ships	Supplementary Table 6
l_{d}	Longitudinal domain lengths of downstream ships	Supplementary Table 6
BCI	Bearing capacity index, representing the navigational capacity of a given waterway	Equation (3)
$\widehat{\mathrm{BC}}_w$	Ascending rank order over all waterways of bearing capacity at waterway w	-
SEI	Socio-economic index, representing transport need driven by socio-economic development	Equation (4)
GDP	Gross domestic product	http://data.un.org/
AIO	Agriculture and industry outputs	http://data.un.org/
POP	Population	http://data.un.org/
$\widehat{\mathrm{GDP}}_w$	Ascending rank orders over all waterways of GDP at waterway *w*	-
$\widehat{\mathrm{AIO}}_w$	Ascending rank orders over all waterways of AIO at waterway *w*	-
$\widehat{\mathrm{POP}}_{i,w}$	Ascending rank orders over all waterways of POP at waterway *w*	-
HDI	Human development index, a metric quantifies life expectancy, educational attainment, and income as a standardized number	http://hdr.undp.org/sites/default/files/2016_human_development_report.pdf

Supplementary Table 13 Interpretation and data source for metrics in the Methods (*continued*)

Metric	Interpretation	Method and Data source
CI	Consistency index, defined as the ratio of transport need to bearing capacity	Equation (5)
TN	Transport need expressed as freight transport volume	Supplementary Table 11
$CAGR_{freight}$	Compound annual growth rate of freight volume	Equation (6)
EC	Elastic coefficient	Supplementary Table 12
$CAGR_{GDP}$	Compound annual growth rate of GDP	https://www.rug.nl/ggdc/historicaldevelopment/maddison/releases/maddison-project-database-2018 https://pardee.du.edu/
ER	Inland waterway exploitation ratio defined as the ratio of the actual to idealized bearing capacity	Equation (7)
IBC	Idealized bearing capacity of waterway, representing the maximum potential of bearing capacity for an inland waterway	Equation (1)
d_w	Minimum waterway maintenance depth for IBC	Equation (8)
d_{dry}	Average water depth in the dry season	http://gaia.geosci.unc.edu/rivers/
EPI	Ecological pressure index, measuring the health of a river ecosystem affected by human activities	Equation (9)
FI	Fragmentation index of dams used for navigational purpose	Equation (10)
FI'	Fragmentation index of all kinds of dams	http://www.riverthreat.net/data.html
WDI	Wetland dis-connectivity index	
FIS	Fraction of impervious surfaces	
FDI	Flow disruption index	

Supplementary Table 13 Interpretation and data source for each metric in the Methods (*continued*)

Metric	Interpretation	Method and Data source
FRI	Fish richness index	https://static-content.springer.com/esm/art%3A10.1007%2Fs10750-012-1242-6/MediaObjects/10750_2012_1242_MOESM2_ESM.txt
PNF	Proportion of non-native fish	
FI_0	Threshold value of FI corresponding to	Fig. 5
WDI_0	Threshold value of WDI when ER approaching 80%	
FIS_0	Threshold value of FIS when ER approaching 80%	
FDI_0	Threshold value of FDI when ER approaching 80%	
FRI_0	Threshold value of FRI when ER approaching 80%	
PNF_0	Threshold value of PNF when ER approaching 80%	
α	Proportional factor, proportion of dams built for navigability purposes	Equation (11)
N_{navi}	Number of dams used for navigation in a basin	http://globaldamwatch.org/grand/
N_{total}	Total number of dams in a basin	
EEI	Eco-efficiency index, defined as the ratio of GDP to ecological footprint	Equation (12)
EF	ecological footprint	https://www.footprintnetwork.org/
SI	Sustainability index, equal to average of S_{CI}, S_{ER}, and $S_{EEI,EPI}$	-
S_{CI}	Score of CI	Equation (13)
S_{ER}	Score of ER	Equation (14)
$S_{EEI,EPI}$	Score of EEI and EPI	Equation (4)

Supplementary References

1. General administration of quality supervision, inspection and quarantine of P. R. C. & Ministry of housing and urban-rural development of the people's republic of China. *Navigation standard of inland waterways.* http://download.mohurd.gov.cn/bzgg/gjbz/GB50139-2014%E5%86%85%E6%B2%B3%E9%80%9A%E8%88%AA%E6%A0%87%E5%87%86.pdf (2014).
2. Zhang, W., Liu, J. A., Sun, H. J., Liu, H. H. & Zhu, M. S. Research on channel transit capacity in Yangtze River deepwater channel. China *Harbour Engineering* **36**, 5-10 (2016). (in Chinese)
3. Li, Y & Zhang, W. On model about ship domain based on stopping sight distance. *Port & Waterway Engineering* 36-40 (2013). DOI:10.16233/j.cnki.issn1002-4972.2014.01.033. (in Chinese)
