# Supplementary material for: Sustainability of global Golden Inland Waterways
Source: Nat Commun. 2020 Mar 25;11:1553. doi: 10.1038/s41467-020-15354-1 (PMC7096509; doi:10.1038/s41467-020-15354-1)
Supplement: Supplementary file 2 — Reporting Summary [file 41467_2020_15354_MOESM2_ESM.pdf]

nature research

Corresponding author(s): Jinren Ni

Last updated by author(s): Mar 4, 2020

Reporting Summary

Nature Research wishes to improve the reproducibility of the work that we publish. This form provides structure for consistency and transparency in reporting. For further information on Nature Research policies, see Authors & Referees and the Editorial Policy Checklist.

Statistics

For all statistical analyses, confirm that the following items are present in the figure legend, table legend, main text, or Methods section.

n/a	Confirmed	
☒	☐	The exact sample size (*n*) for each experimental group/condition, given as a discrete number and unit of measurement
☒	☐	A statement on whether measurements were taken from distinct samples or whether the same sample was measured repeatedly
☒	☐	The statistical test(s) used AND whether they are one- or two-sided<br>*Only common tests should be described solely by name; describe more complex techniques in the Methods section.*
☒	☐	A description of all covariates tested
☒	☐	A description of any assumptions or corrections, such as tests of normality and adjustment for multiple comparisons
☐	☒	A full description of the statistical parameters including central tendency (e.g. means) or other basic estimates (e.g. regression coefficient) AND variation (e.g. standard deviation) or associated estimates of uncertainty (e.g. confidence intervals)
☒	☐	For null hypothesis testing, the test statistic (e.g. *F*, *t*, *r*) with confidence intervals, effect sizes, degrees of freedom and *P* value noted<br>*Give P values as exact values whenever suitable.*
☐	☒	For Bayesian analysis, information on the choice of priors and Markov chain Monte Carlo settings
☒	☐	For hierarchical and complex designs, identification of the appropriate level for tests and full reporting of outcomes
☒	☐	Estimates of effect sizes (e.g. Cohen's *d*, Pearson's *r*), indicating how they were calculated

Our web collection on statistics for biologists contains articles on many of the points above.

Software and code

Policy information about availability of computer code

Data collection	No software was used.
Data analysis	ArcMap10.2 and python code were used to analyse the data in this study.

For manuscripts utilizing custom algorithms or software that are central to the research but not yet described in published literature, software must be made available to editors/reviewers. We strongly encourage code deposition in a community repository (e.g. GitHub). See the Nature Research guidelines for submitting code & software for further information.

Data

Policy information about availability of data

All manuscripts must include a data availability statement. This statement should provide the following information, where applicable:

- Accession codes, unique identifiers, or web links for publicly available datasets
- A list of figures that have associated raw data
- A description of any restrictions on data availability

The data of physical and socio-economic characteristics of global large inland waterways at reach scale are available at figshare (DOI: 10.6084/m9.figshare.11653281). Basin-scale data related to inland waterways reported in this paper are provided in Supplementary Information file and Source Data file. All other data, including river networks, basin boundaries, GDP, agriculture and industry outputs, population, river depths, dam distribution, ecological indices and ecological footprint are publicly available as described in the Methods. The source data underlying Figs 2–6 and Supplementary Figs 1, 2, 4, 5 and 7–17 are provided as a Source Data file.

Field-specific reporting

Please select the one below that is the best fit for your research. If you are not sure, read the appropriate sections before making your selection.

☐ Life sciences ☐ Behavioural & social sciences ☒ Ecological, evolutionary & environmental sciences

For a reference copy of the document with all sections, see nature.com/documents/nr-reporting-summary-flat.pdf

Ecological, evolutionary & environmental sciences study design

All studies must disclose on these points even when the disclosure is negative.

Study description	This study identified 34 golden inland waterways (GIWs) each with considerable bearing capacity and transport need, based on datasets from 66 large rivers (basin area > 100,000 km2) worldwide. We further estimated the sustainability of global GIWs in 2015 and 2050 in terms of regional development, exploitation ratio, and ecosystem status.
Research sample	34 large inland waterways identified from 66 large rivers of basin area larger than 100,000 km2
Sampling strategy	66 large rivers with basin area > 100,100 km2 were selected from HYDROSHEDS and PKU river network datasets. The selected 66 large river basin have covered about half of the word's total land area.<br>34 large inland waterways were identified from 66 large rivers based on the bearing capacity and transport need resulting from socio-economic development of the associated river basins. The selected 34 large inland waterways have contained the most famous and concerned large inland waterways.
Data collection	All data used in this study were collected from publicly available sources including open data websites, government agencies websites, and literatures by Xiabin Chen and Yichu Wang.
Timing and spatial scale	This study is global scale. The time scale of data collected and projected in this study ranges from 1860 to 2050.
Data exclusions	No data were excluded from the analyses.
Reproducibility	All attempts to repeat the experiment were successful.
Randomization	Nine types of large waterways were classified based on a bearing capacity index, BCI , determined as the basin-averaged inland waterway bearing capacity; and a socio-economic index, SEI established from gross domestic product, agriculture and industry outputs, and population. BCI and SEI were each divided into three levels (small S; middle M; and large L) at threshold values of 0.33 and 0.67, which were primarily determined according to the significance of cost-effective advantage of inland waterway transport and the level of human development of the river basin of interest. Consequently, nine basic patterns of inland waterway were classified as L-L, L-M, L-S, M-L, M-M, M-S, S-L, S-M, and S-S (the letters prior and post the hyphen denote the level of BCI and SEI for inland waterways, respectively).
Blinding	It is not a experiment-based study.

Did the study involve field work? ☐ Yes ☒ No

Reporting for specific materials, systems and methods

We require information from authors about some types of materials, experimental systems and methods used in many studies. Here, indicate whether each material, system or method listed is relevant to your study. If you are not sure if a list item applies to your research, read the appropriate section before selecting a response.

Materials & experimental systems

n/a	Involved in the study
☒	☐ Antibodies
☒	☐ Eukaryotic cell lines
☒	☐ Palaeontology
☒	☐ Animals and other organisms
☒	☐ Human research participants
☒	☐ Clinical data

Methods

n/a	Involved in the study
☒	☐ ChIP-seq
☒	☐ Flow cytometry
☒	☐ MRI-based neuroimaging
